# 5-HT recruits distinct neurocircuits to inhibit hunger-driven and non-hunger-driven feeding

**DOI:** 10.1038/s41380-021-01220-z

**Published:** 2021-07-21

**Authors:** Yanlin He, Xing Cai, Hailan Liu, Krisitine M. Conde, Pingwen Xu, Yongxiang Li, Chunmei Wang, Meng Yu, Yang He, Hesong Liu, Chen Liang, Tingting Yang, Yongjie Yang, Kaifan Yu, Julia Wang, Rong Zheng, Feng Liu, Zheng Sun, Lora Heisler, Qi Wu, Qingchun Tong, Canjun Zhu, Gang Shu, Yong Xu

**Affiliations:** 1 Children’s Nutrition Research Center, Department of Pediatrics, Baylor College of Medicine, Houston, TX USA; 2 Guangdong Laboratory of Lingnan Modern Agriculture and Guangdong Province Key Laboratory of Animal Nutritional Regulation, College of Animal Science, South China Agricultural University, Guangdong, China; 3 Departments of Pharmacology, University of Texas Health at San Antonio, San Antonio, TX USA; 4 Department of Molecular and Cellular Biology, Baylor College of Medicine, Houston, TX USA; 5 Department of Medicine, Division of Diabetes, Endocrinology and Metabolism, Baylor College of Medicine, Houston, TX USA; 6 Rowett Institute, University of Aberdeen, Foresterhill, Aberdeen UK; 7 Brown Foundation Institute of Molecular Medicine, University of Texas Health Science Center at Houston, Houston, TX USA; 8 Present Address: Pennington Biomedical Research Center, Brain Glycemic and Metabolism Control Department, Louisiana State University, Baton Rouge, LA USA; 9 Present Address: State Key Laboratory of Genetic Resources and Evolution, Kunming Institute of Zoology, Chinese Academy of Sciences, Kunming, Yunnan China; 10 Present Address: Division of Endocrinology, Department of Medicine, The University of Illinois at Chicago, Chicago, IL USA

**Keywords:** Physiology, Neuroscience

## Abstract

Obesity is primarily a consequence of consuming calories beyond energetic requirements, but underpinning drivers have not been fully defined. 5-Hydroxytryptamine (5-HT) neurons in the dorsal Raphe nucleus (5-HT^DRN^) regulate different types of feeding behavior, such as eating to cope with hunger or for pleasure. Here, we observed that activation of 5-HT^DRN^ to hypothalamic arcuate nucleus (5-HT^DRN^ → ARH) projections inhibits food intake driven by hunger via actions at ARH 5-HT_2C_ and 5-HT_1B_ receptors, whereas activation of 5-HT^DRN^ to ventral tegmental area (5-HT^DRN^ → VTA) projections inhibits non-hunger-driven feeding via actions at 5-HT_2C_ receptors. Further, hunger-driven feeding gradually activates ARH-projecting 5-HT^DRN^ neurons via inhibiting their responsiveness to inhibitory GABAergic inputs; non-hunger-driven feeding activates VTA-projecting 5-HT^DRN^ neurons through reducing a potassium outward current. Thus, our results support a model whereby parallel circuits modulate feeding behavior either in response to hunger or to hunger-independent cues.

## Introduction

The prevalence of obesity has substantially increased since the 1950s. Strategies to combat obesity and related comorbidities, including type 2 diabetes, cardiovascular disease, and some types of cancer are urgently required. Obesity is primarily a consequence of the consumption of more food than the body requires; calories that are consequently stored as fat. For some individuals, overeating is primarily driven by hunger (a state of nutritional deficit), whereas others overeat in the absence of hunger [1, 2]. A better understanding of the neurobiological mechanisms for these two types of feeding behaviors is essential to develop rational precision medicines to more effectively treat the complex etiology of obesity.

The brain serotonin (5-hydroxytryptamine, 5-HT) system plays critical roles in the regulation of feeding. Brain 5-HT is primarily synthesized by neurons in the dorsal Raphe nucleus (DRN) in the midbrain, which projects to other midbrain regions and the hypothalamus [3]. Hunger decreases 5-HT release from the DRN, while satiation increases it [4], suggesting that dynamic 5-HT bioavailability may participate in the physiological regulation of feeding behavior. Indeed, d-fenfluramine (d-Fen), a pharmacological agent that increases 5-HT content [5], showed a potent anorexigenic activity in rodents and humans [6–8]. Conversely, treatments that suppress central 5-HT signals produce hyperphagia and weight gain [9–12]. The brain 5-HT system has been an attractive target for anti-obesity therapies. For example, d-Fen, used in combination with phentermine (as Fen/Phen), was widely prescribed obesity medication in the 1990s. The therapeutic benefit of Fen/Phen was established to be mediated primarily via 5-HT_2C_ receptors (5-HT_2C_Rs); the 5-HT_2C_R agonist lorcaserin was clinically used to treat human obesity [13]. Recent evidence indicates that lorcaserin not only inhibits food intake triggered by hunger [14, 15], but also inhibits the consumption of high palatable foods in the absence of hunger [15, 16]. These results suggest that the brain 5-HT system can inhibit both hunger-driven and non-hunger-driven feeding. Although the Fen/Phen regimen [17] and lorcaserin [18] were no longer used in the clinic due to nonspecific adverse effects, better understanding about how the brain 5-HT circuitry regulates various types of feeding behaviors will provide a necessary framework to develop novel strategies to combat the obesity pandemic.

The arcuate nucleus of hypothalamus (ARH) and the ventral tegmental area (VTA) are two brain regions implicated in the control of energy homeostasis [19, 20] and motivated behaviors [21–24], respectively. Here, we characterized the neurotransmission between 5-HT^DRN^ neurons and distinct types of neurons in the ARH and VTA, and further examined the effects of the 5-HT^DRN^ → ARH and 5-HT^DRN^ → VTA projections on hunger-driven and non-hunger-driven feeding. In addition, we assessed the dynamic changes of 5-HT^DRN^ neuron activities during the course of hunger-driven and non-hunger-driven feeding, and delineated distinct ionic mechanisms by which 5-HT^DRN^ neurons are regulated.

## Materials and methods

### Mice

TPH2-CreER mice were purchased from Jackson Laboratory (#016584) that express tamoxifen-inducible Cre recombinase selectively in 5-HT neurons, as we validated previously [25]. Dopamine transporter (DAT)-CreER mice were purchased from Jackson Laboratory (#016583) that express tamoxifen-inducible Cre recombinase selectively in dopamine (DA) neurons, as we validated previously [25]. Tamoxifen-inducible POMC-CreER mice were obtained from Dr. Joel Elmquist lab [26] and has been previously validated in the lab [27, 28]. All these Cre lines, as well as AgRP-Cre mice [29], were bred with Rosa26-LSL-tdTOMATO mice [30] (Jackson Laboratory, #007905), to generate reporter mice, and tdTOMATO signals were examined throughout the brain to confirm the expected Cre expression pattern. We also crossed TPH2-CreER onto POMC-CreER/Rosa26-LSL-tdTOMATO, AgRP-Cre/Rosa26-LSL-tdTOMATO, or DAT-CreER/Rosa26-LSL-tdTOMATO mice to generate compound Cre lines for CRACM studies, as described below. Some TPH2-CreER mice were used for optogenetic and fiber photometry studies as outlined below. In addition, some TPH2-CreER/Rosa26-LSL-tdTOMATO mice, as well as C57Bl6j mice (purchased from the mouse facility of Baylor College of Medicine), were used for retrograde tracing and electrophysiology studies. Further, we crossed TPH2-CreER allele onto γ2^fl/fl^ mice [31] or SK3^fl/fl^ mice [32] (Jackson Laboratory, #019083) to generate mice lacking γ2 or SK3 selectively in 5-HT neurons; littermate γ2^fl/fl^ or SK3^fl/fl^ mice were used as controls. All the breeders have been backcrossed to C57Bl6j background for >12 generations. Mice were housed in a temperature-controlled environment in groups of two to five at 22–24 °C, using a 12 h light/12 h dark cycle. All mice were fed standard chow (6.5% fat, #2920, Harlan-Teklad, Madison, WI) ad libitum, unless described otherwise. Water was provided ad libitum.

### Neurotracing

To determine whether 5-HT^DRN^ neurons project to the ARH and VTA, 12-week-old TPH2-CreER mice were anesthetized by isoflurane and received stereotaxic injections of Cre-dependent adeno-associated virus (AAV) expressing wheat germ agglutinin (WGA)-zsGreen (AAV9-CBA-DIO-WGA-zsGreen, 7.6 × 10^12^ GC/ml) into the DRN (200 nl, 4.65 mm posterior, 0 mm lateral, and 3.6 mm ventral to the Bregma). These mice also received an i.p. injection of tamoxifen (0.2 mg/g) to induce Cre activity. One week after injections, mice were perfused with 10% formalin, and brain sections were cut at 25 μm (five series). One series of the sections were blocked (3% normal donkey serum) for 1 h, incubated with goat anti-WGA antibody (1:1000; AS-2024, Vector Laboratories) on shaker at 4 °C for overnight, followed by the donkey anti-goat AlexaFluor 488 (1:200, A11055, Invitrogen) for 2 h. Slides were cover-slipped and analyzed using a Leica DM5500 fluorescence microscope with OptiGrid structured illumination configuration.

To further confirm the projections of 5-HT^DRN^ neurons project to the ARH and VTA, 12-week-old C57Bl6j mice were anesthetized by isoflurane and received stereotaxic injections of Red RetroBeads and Green RetroBeads into the ARH (bilateral 200 nl/site, 1.7 mm posterior, 0.25 mm lateral, and 5.9 mm ventral to the Bregma) and VTA (bilateral 200 nl/site, 3 mm posterior, 0.5 mm lateral, and 4.5 mm ventral to the Bregma), respectively. Four weeks after, the mice were perfused as above. Brain sections were then subjected to immunofluorescent staining for 5-HT. Briefly, sections were incubated with primary rabbit anti-5-HT antibody (1:10,000, 20080, ImmunoStar) overnight, followed by the goat anti-rabbit AlexaFluor 405 (1:500, A31556, Invitrogen) for 1.5 h. The sections were mounted on glass slides and fluorescence images were analyzed using a Leica DM5500 fluorescence microscope with OptiGrid structured illumination configuration. The DRN neurons with 5-HT immunoreactivity co-labeled by Red RetroBeads, by Green RetroBeads, or by both beads were counted in ten mice.

### CRACM

In order to examine the neurotransmissions between 5-HT^DRN^ neurons and POMC^ARH^, AgRP^ARH^, or DA^VTA^ neurons, we performed the channelrhodopsin-2 (ChR2)-assisted circuit mapping (CRACM), similarly as we described before [33]. Three different compound mouse strains were used: TPH2-CreER/POMC-CreER/Rosa26-LSL-tdTOMATO, TPH2-CreER/AgRP-Cre/Rosa26-LSL-tdTOMATO, and TPH2-CreER/DAT-CreER/Rosa26-LSL-tdTOMATO. These mice were anesthetized by isoflurane and received stereotaxic injections of Ad-iN/WED (100 nl) and AAV-EF1α-DIO hChR2(H134R)-enhanced yellow fluorescent protein (EYFP; 100 nl) into the DRN. Tamoxifen (0.2 mg/g, i.p.) was injected into these mice to induce Cre activities. After a 4-week recovery, mice were sacrificed and unfixed brain slices (containing both the DRN and ARH, or DRN and VTA, 120 µm in thickness) were prepared from these mice. These brain slices were subjected to fluorescent microscopy to visualize and quantify WGA(+), tdTOMATO(+), and WGA(+)/tdTOMATO(+) neural somas in the ARH or VTA. Notably, EYFP expressed by the AAV-EF1α-DIO hChR2(H134R)-EYFP shares overlapping spectra with GFP. However, EYFP will only label the fibers and terminals in the ARH or VTA, but will not cross the synapse to label the somas of downstream neurons. Thus, the GFP-labeled somas within the ARH or VTA must be filled by WGA-GFP fusion proteins. About 500–600 neural somas were counted from each mouse and three to five mice were included for each strain. These brain slices were also used to perform electrophysiological recordings, as described below.

Under the voltage-clamp mode, light-evoked excitatory postsynaptic currents (eEPSCs) or inhibitory postsynaptic currents (eIPSCs) were recorded in WGA(+)/tdTOMATO(+) neurons in the ARH or VTA in response to 473 nm laser blue light (10 Hz, 10 ms pulse, 0.2 mW; MBL-FN-473, CNI LASER). The eEPSCs were recorded in whole-cell voltage-clamp mode, by holding the membrane potential at Vh = −60 mV. The pipette solution containing 125 mM CsCH_3_SO_3_, 10 mM CsCl, 5 mM NaCl, 2 mM MgCl_2_, 1 mM EGTA, 10 mM HEPES, 5 mM (Mg)ATP, and 0.3 mM (Na)_2_GTP (pH 7.3 with NaOH). The eIPSCs were recorded in whole-cell voltage-clamp mode, by holding the membrane potential at Vh = −70 mV. The CsCl-based pipette solution containing of 140 mM CsCl, 10 mM HEPES, 5 mM MgCl_2_, 1 mM BAPTA, 5 mM (Mg)ATP, and 0.3 mM (Na)_2_GTP (pH 7.30 adjusted with NaOH; 295 mOsm/kg). TTX (1 μM) and 4-AP (400 μM) were added to the artificial cerebrospinal fluid (aCSF), in order to confirm the response was monosynaptic responses. eEPSCs were recorded when 50 μM bicuculline was applied in the bath solution; after an eEPSC was detected, 30 μM D-AP5 and 30 μM CNQX were used to confirm the current was mediated by glutamate receptors. eIPSCs were recorded when 30 μM D-AP5 and 30 μM CNQX were applied in the bath solution; after an eIPSC was detected, 50 μM bicuculline was used to confirm the current was mediated by GABA_A_ receptor. Under the current-clamp mode, 30 μM D-AP5, 30 μM CNQX, and 50 μM bicuculline were applied in the bath solution to block the glutamate and GABA inputs; action potential firing frequency and resting membrane potential were recorded in response to 473 nm blue light (20 Hz, 10 ms pulse, 0.2 mW) in the absence or presence of 100 μM SB242084 (a 5-HT_2C_R antagonist) [15], or 10 μM SB224289 (a 5-HT_1B_R antagonist) [34]. Note that some neurons did not spontaneously fire in the presence of D-AP5, CNQX, and bicuculline cocktail, and therefore were not included in the CRACM experiments.

### Optogenetics and hunger-driven or non-hunger-driven feeding

TPH2-CreER mice (12 weeks of age) were anesthetized with isoflurane and received stereotaxic injections of Cre-dependent AAV expressing ChR2-YFP (AAV-EF1α-DIO hChR2(H134R)-YFP, 6.2 × 10^12^ VP/ml) into the DRN (200 nl; 4.65 mm posterior, 0 mm lateral, and 3.6 mm ventral to the Bregma). Simultaneously, an optic fiber (0.2 mm in diameter with a numerical aperture of 0.22) was implanted to target the ARH (1.7 mm posterior, 0.25 mm lateral, and 5.8 mm ventral to the Bregma) or the VTA (3 mm posterior, 0.5 mm lateral, and 4.3 mm ventral to the Bregma). Four weeks after, mice were subjected to hunger-driven feeding paradigm. Briefly, mice were singly housed with chow diet ad libitum. Mice were then fasted overnight, and chow diet was provided back to the cage at 10 a.m. next morning. Blue (473 nm, 10 ms per pulse, 20 pulses per second) or yellow light (589 nm, 10 ms per pulse, 20 pulses per second) pulses were delivered to the ARH or VTA through the optic fiber for one hour (10–11 a.m.). To avoid damages associated with optic stimulations, we provide 10 ms per pulse at 20 pulses per second (200 ms per second) leaving the majority of the period unstimulated. Light power was applied at 10 mW for both photostimulation and photoinhibition to reach appropriate light power exiting the fiber tip in the brain for activation and inhibition, respectively. Food (chow) intake was measured during the stimulation period and also at 2-h time point. The same mice received blue or yellow light pulses in different trials in a crossover fashion, with a 1-week interval. The order of blue and yellow light stimulation was randomized to avoid potential sequence effects.

After the hunger-driven feeding, the same mice were then subjected to the non-hunger-driven feeding paradigm. One week before the experiment, chow-fed mice were presented with a high-fat diet (HFD) pellets (40% fat; TD.95217, Harlan) for 1 day to avoid neophobia, and then they were maintained on chow diet ad libitum. On the experimental day, at 10 a.m., the HFD was provided to the cage, together with chow diet. Photostimulations (blue or yellow light pulses) were delivered through the optic fiber for 1 h (10–11 a.m.), similarly as described above. Food (chow and HFD) intake was monitored during the stimulation period and also at 2.5-h time point.

### Optogenetics and real-time place preference test

Mice were subjected to the procedure room 1 h before the start of each test and remained in the same room throughout the test. The experimental box contained two chambers that are connected by a doorway. The two chambers were 50 × 50 × 25 cm for each with black pexiglass wall and white pexiglass floor, and connected by an opening (12.5 cm) in the center. In each trial, mice were allowed to explore the two chambers for half an hour (during the light cycle). After acclimation, the time spent in each chamber was recorded and analyzed for chamber preference. To validate accurate and sufficient infection of ChR2-EYFP in 5-HT^DRN^ neurons, all mice were perfused with 10% formalin. Brain sections were cut at 25 μm (five series) and subjected to histological validation. Only those mice with EYFP in the DRN and the fiber tract in the ARH or VTA were included in analyses.

### Electrophysiology

Twelve-week-old TPH2-CreER/Rosa26-LSL-tdTOMATO mice were anesthetized by isoflurane and received stereotaxic injections of Green RetroBeads and Purple RetroBeads into the ARH and VTA (bilateral, 200 nl/site), respectively. Four weeks after, the mice were subjected to hunger-driven or non-hunger-driven feeding paradigm as described above. For mice undergoing hunger-driven feeding, mice were sacrificed when fed ad libitum, after a 24-h fasting, or after a 24-h fasting followed by a 2-h refeeding. For non-hunger-driven feeding, mice were sacrificed immediately before or 1 h after they started the non-hunger-driven feeding. Briefly, mice were deeply anesthetized with isoflurane at aforementioned time points and transcardially perfused with a modified ice-cold sucrose-based cutting solution (pH 7.3) containing 10 mM NaCl, 25 mM NaHCO_3_, 195 mM sucrose, 5 mM glucose, 2.5 mM KCl, 1.25 mM NaH_2_PO_4_, 2 mM Na-pyruvate, 0.5 mM CaCl_2_, and 7 mM MgCl_2_, bubbled continuously with 95% O_2_ and 5% CO_2_ (Ren et al., 2012). The mice were then decapitated, and the entire brain was removed and immediately submerged in the cutting solution. Slices (250 µm) were cut with a Microm HM 650 V vibratome (Thermo Scientific). Brain slices containing the DRN were obtained for each animal (Bregma −2.06 mm to −1.46 mm; interaural 1.74–2.34 mm). The slices were recovered for 1 h at 34 °C and then maintained at room temperature in aCSF (pH 7.3) containing 126 mM NaCl, 2.5 mM KCl, 2.4 mM CaCl_2_, 1.2 mM NaH_2_PO_4_, 1.2 mM MgCl_2_, 5.0 mM glucose, and 21.4 mM NaHCO_3_) saturated with 95% O_2_ and 5% CO_2_ before recording.

Slices were transferred to a recording chamber and allowed to equilibrate for at least 10 min before recording. The slices were superfused at 34 °C in oxygenated aCSF at a flow rate of 1.8–2 ml/min. tdTOMATO(+) neurons, co-labeled with Green or Purple RetroBeads, in the DRN were visualized using epifluorescence and IR-DIC imaging on an upright microscope (Eclipse FN-1, Nikon) equipped with a movable stage (MP-285, Sutter Instrument). Patch pipettes with resistances of 3–5 MΩ were filled with intracellular solution (pH 7.3) containing 128 mM K-gluconate, 10 mM KCl, 10 mM HEPES, 0.1 mM EGTA, 2 mM MgCl_2_, 0.05 mM Na-GTP, and 0.05 mM Mg-ATP. Recordings were made using a MultiClamp 700B amplifier (Axon Instrument), sampled using Digidata 1440 A and analyzed offline with pClamp 10.3 software (Axon Instruments). Series resistance was monitored during the recording, and the values were generally <10 MΩ and were not compensated. The liquid junction potential was +12.5 mV, and was corrected after the experiment. Data were excluded if the series resistance increased dramatically during the experiment or without overshoot for action potential. Currents were amplified, filtered at 1 kHz, and digitized at 20 kHz. The current-clamp mode was engaged to measure neural firing frequency and resting membrane potential. The values for resting membrane potential and firing frequency were averaged within 2-min bin.

A voltage-clamp protocol was used to record the small conductance Ca^2+^-activated K^+^ (SK) current as we did before [35, 36]. Briefly, patch pipettes with resistances of 3–5 MΩ were filled with intracellular solution (pH 7.3) containing 128 mM K-gluconate, 10 mM KCl, 10 mM HEPES, 0.1 mM EGTA, 2 mM MgCl_2_, 0.05 mM Na-GTP, and 0.05 mM Mg-ATP. The SK tail current was evoked by a 100 ms depolarizing pulse from a holding potential −60 mV to 0 mV and then back to −60 mV. To measure miniature EPSC (mEPSC), the internal recording solution contained 125 mM CsCH_3_SO_3_, 10 mM CsCl, 5 mM NaCl, 2 mM MgCl_2_, 1 mM EGTA, 10 mM HEPES, 5 mM (Mg)ATP, and 0.3 mM (Na)2GTP (pH 7.3 with NaOH) [37]. The mEPSCs were recorded in whole-cell voltage-clamp mode, by holding the membrane potential at Vh = −60 mV in the presence of 1 μM TTX and 50 μM bicuculline. The miniature IPSCs (mIPSCs) were recorded in whole-cell voltage-clamp mode by holding the membrane potential at Vh = −70 mV. The CsCl-based pipette solution contains 140 mM CsCl, 10 mM HEPES, 5 mM MgCl_2_, 1 mM BAPTA, 5 mM (Mg)ATP, and 0.3 mM (Na)_2_GTP (pH 7.30 adjusted with NaOH; 295 mOsm/kg). The mIPSCs were recorded in the presence of 1 μM TTX, 30 μM D-AP5, and 30 μM CNQX [38]. Frequency and peak amplitude were measured using the Mini Analysis program (Synaptosoft Inc.).

### Validation of γ2 or SK3 deletion

To validate selective deletion of γ2 from 5-HT neurons, γ2-TPH2-KO mice and control littermates were perfused with 10% formalin, and brain sections were cut at 25 μm (five series). These brain sections were subjected to dual immunofluorescent staining for γ2 and 5-HT. Briefly, sections were incubated with primary rabbit anti-γ2 antibody (1:100, NB300-190, Novus Biologicals) and primary goat anti-5-HT antibody (1:5000, 20079, ImmunoStar) overnight, followed by the donkey anti-rabbit AlexaFluor 594 (1:200, A21207, Invitrogen) and donkey anti-goat AlexaFluor 488 (1:500, A11055, Invitrogen) for 1.5 h. γ2 Immunoreactivity (red) and 5-HT immunoreactivity (green) fluorescence signals were analyzed using a Leica DM5500 fluorescence microscope with OptiGrid structured illumination configuration.

We used real-time RT-qPCR to examine levels of SK3 mRNAs in the DRN from control vs. SK3-TPH2-KO mice. To this end, the mouse brains were removed, the DRN was quickly dissected, and subjected to RNA extraction and reverse transcription using the qSript XLT cDNA SuperMix (Quanta Biosciences, 95161-025), according to the manufacturer’s instruction. Briefly, 2 μl of qSript XLT cDNA SuperMix was added to 8 μl of each sample, and 10 μl of this RT mixture were used for cDNA synthesis (25 °C for 10 min, 42 °C for 60 min, and 85 °C for 5 min). The cDNA samples were amplified on a CFX384 Real-Time System (Bio-Rad) using SsoADV SYBR Green Supermix (Bio-Rad). Results were normalized against the expression of house-keeping gene. Primer sequences were for cyclophilin: forward-TGGAGAGCACCAAGACAGACA and reverse-TGCCGGAGTCGACAATGAT; for SK3: forward-TGTTGCACTCTTCTCCCACG and reverse-GGTCATTGAGATTTAGCTGGCT.

### Fiber photometry

For the fiber photometry experiments, TPH2-CreER mice (12 weeks of age) were anesthetized by isoflurane and received stereotaxic injections of pAAV9.Syn.Flex.GCaMP6m WPRE.SV40 (200 nl/site, 3 × 10^9^ VP/ml) into the DRN. During the same surgery, an optical fiber (fiber: core = 400 μm; 0.48 NA; M3 thread titanium receptacle; Doric Lenses) was implanted over the DRN (4.65 mm posterior, 0 mm lateral, and 3.30 mm ventral to the Bregma, based on Franklin and Paxinos Mouse Brain Atlas). Fibers were fixed to the skull using dental acrylic and mice were allowed 3 weeks for recovery before acclimatization investigator handling for 1 week before experiments.

The fiber photometry recordings started 4–6 weeks after surgeries to allow for adequate recovery and GCaMP6m expression to stabilize. All recordings were done in the home cage of the singly housed mice that were subjected to either hunger-driven or non-hunger-driven feeding paradigm as described above. Mice were allowed to adapt to the tethered patchcord for 2 days prior to experiments and given 5 min to acclimate to the tethered patchcord prior to any recording. Recordings were performed during the first 5 min of each 30-min bin, to avoid photobleaching. Continuous <20 μW blue LED at 465 nm and UV LED at 405 nm served as excitation light sources, driven by a multichannel hub (Doric Lenses), modulated at 211 and 330 Hz, respectively. A 465 nm excites calcium-dependent fluorescence from GCaMP6m protein, providing a measure of neuron activity. A 405 nm excites calcium-independent fluorescence from GCaMP6m protein and serves as a control for movement and bleaching artifacts. The light was delivered to a filtered minicube (FMC5, Doric Lenses) before connecting through optic fibers to a rotary joint (FRJ 1 × 1, Doric Lenses) to allow for movement. GCaMP6m calcium GFP signals and UV autofluorescent signals were collected through the same fibers back to the dichroic ports of the minicube into a femtowatt silicon photoreceiver (2151, Newport). The digital signals were then amplified, demodulated, and collected through a lock-in amplifier (RZ5P, Tucker-Davis Technologies) [39]. The fiber photometry data was down sampled to 8 Hz. To align acute neural responses (in seconds) to each eating bout, animals’ behavior were also simultaneously recorded with a camera. We derived the values of GCaMP6 fluorescence change (Δ*F*/*F*_0_) by calculating (*F*_465_ − *F*_0_)/*F*_0_, where *F*_0_ is the baseline fluorescence signal 2 s prior to the onset of each eating bout [40]. The *F*_405_ channel is used as an isosbestic fluorescence channel; we derived the values of isosbestic fluorescence change (Δ*F*/*F*_0_) by calculating (*F*_405_ − *F*_0_)/*F*_0_, where *F*_0_ is the baseline fluorescence of the *F*_405_ channel signal 2 s prior to the onset of each eating bout. To evaluate the slow changes in neural activity (every 30 min), we derived the values of fluorescence change (Δ*F*/*F*_*n*_) by calculating (*F*_465_ − *F*_405_)/*F*_405_, in order to minimize the interference of bleaching artifacts [41].

### Statistical analyses

The minimal sample size was predetermined by the nature of experiments. The data are presented as mean ± SEM and/or individual data points. Statistical analyses were performed using GraphPad Prism to evaluate normal distribution and variations within and among groups. Methods of statistical analyses were chosen based on the design of each experiment and are indicated in figure legends. *P* < 0.05 was considered to be statistically significant. Note that both male and female animals were included; since we did not observe sex differences, data from both sexes were pooled for statistical analyses.

### Study approval

Care of all animals and procedures were approved by the Baylor College of Medicine Institutional Animal Care and Use Committee.

## Results

### 5-HT^DRN^ neurons project to the ARH and VTA

To examine whether 5-HT^DRN^ neurons project to the ARH and VTA, we stereotaxically injected AAV-DIO-WGA-zsGreen into the DRN of TPH2-CreER mice (with tamoxifen induction) to selectively express WGA-zsGreen in 5-HT^DRN^ neurons (Fig. S1A, B). WGA is an anterograde trans-synaptic tracer, which can label the somas of downstream neurons that are immediately innervated by 5-HT^DRN^ terminals [42]. We observed abundant WGA-zsGreen-labeled cell bodies in the ARH and VTA (Fig. S1C, D), confirming that 5-HT^DRN^ neurons project to both of these brain regions. Next, we sought to determine whether the ARH and VTA receive projections from the same or segregated 5-HT^DRN^ neurons. To this end, we stereotaxically injected red and green retrograde tracers (RetroBeads) into the ARH and VTA, respectively, in the same mice (Fig. S1E, F). As expected, we detected both red and green RetroBeads in the DRN, which were colocalized with 5-HT immunoreactivity (Fig. S1G), confirming that 5-HT^DRN^ neurons project to the ARH and/or VTA. Consistent with the broad 5-HT^DRN^-originated projections throughout the brain regions other than the ARH and VTA [43, 44], we noted that the majority of 5-HT^DRN^ neurons were not labeled by retrograde beads injected into the ARH and VTA (Fig. S1G, H). Thus, only a small subset of 5-HT^DRN^ neurons provide projections to the ARH and/or VTA (as co-labeled by either green beads or red beads). Within this 5-HT^DRN^ subset projecting to the ARH and/or VTA, only 13.27% have collateral projections to both regions (co-labeled by both green and red beads), while the majority project to either ARH only (33.71%) or VTA only (53.02%), indicating that ARH- and VTA-projecting 5-HT^DRN^ neurons are largely segregated (Fig. S1H).

### 5-HT^DRN^ → ARH circuit inhibits hunger-driven feeding but not non-hunger-driven feeding

**Fig. 1:**
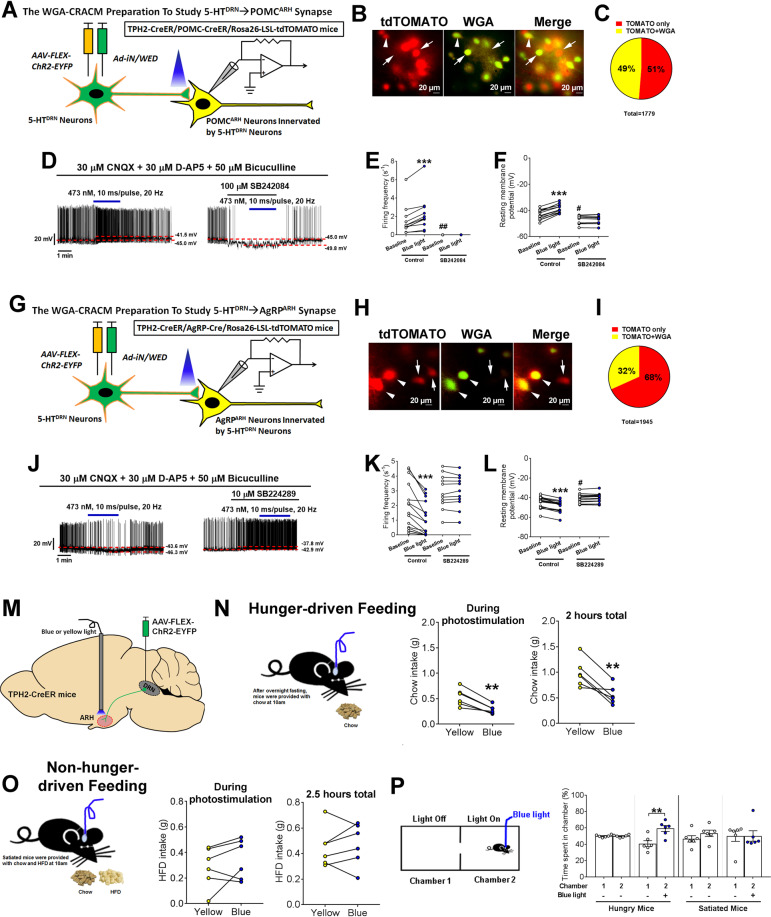
5-HT^DRN^ → ARH circuit inhibits hunger-driven feeding but not non-hunger-driven feeding. **A** Recordings in 5-HT^DRN^-innervated POMC^ARH^ neurons [tdTOMATO(+)WGA(+)] in response to photostimulation of ChR2-labeled 5-HT^DRN^-originated fibers within the ARH. **B** Representative images showing ARH neurons labeled by tdTOMATO, WGA, or both. Scale bars = 20 µm. Arrows point to the single-labeled neurons and the arrowhead points to a double-labeled neuron. **C** The composition of tdTOMATO-labeled POMC^ARH^ neurons that are tdTOMATO(+) only and tdTOMATO(+)WGA(+). A total of 1779 POMC^ARH^ neurons were counted from three mice. **D** Representative action potential traces in response to blue light pulses (473 nm, 10 ms/pulse, 20 Hz) in the absence or the presence of 100 µM SB242084 in a bath solution containing 30 µM CNQX, 30 µM D-AP5, and 50 µM bicuculline. **E**, **F** Firing frequency (**E**) and resting membrane potential (**F**) at the baseline or after blue light stimulation in the absence or the presence of 100 µM SB242084. Results are shown as individual data points. ***P < 0.001 between blue light vs. baseline; #*P* < 0.05 and ##*P* < 0.01 between control and SB242084 in two-way ANOVA analyses followed by Sidak post hoc test (*N* = 10–14 neurons from three mice per group). **G** Recordings in 5-HT^DRN^-innervated AgRP^ARH^ neurons [tdTOMATO(+)WGA(+)] in response to photostimulation of ChR2-labeled 5-HT^DRN^-originated fibers within the ARH. **H** Representative images showing ARH neurons labeled by tdTOMATO, WGA, or both. Scale bars = 20 µm. Arrows point to the single-labeled neurons and arrowheads point to double-labeled neurons. **I** The composition of tdTOMATO-labeled AgRP^ARH^ neurons that are tdTOMATO(+) only and tdTOMATO(+)WGA(+). A total of 1945 AgRP^ARH^ neurons were counted from three mice. **J** Representative action potential traces in response to blue light pulses (473 nm, 10 ms/pulse, 20 Hz) in the absence or the presence of 10 µM SB224289 in a bath solution containing 30 µM CNQX, 30 µM D-AP5, and 50 µM bicuculline. **K**, **L** Firing frequency (**K**) and resting membrane potential (**L**) at the baseline or after blue light stimulation in the absence or the presence of 10 µM SB224289. Results are shown as individual data points. ***P < 0.001 between blue light vs. baseline; #*P* < 0.05 between control and SB224289 in two-way ANOVA analyses followed by Sidak post hoc test (*N* = 11–15 neurons from three mice per group). **M** Schematic experimental strategy using optogenetics to activate the 5-HT^DRN^ → ARH projections. **N** The experimental paradigm to measure hunger-driven feeding. Chow intake during photostimulation and during the total 2-h period in mice with the 5-HT^DRN^ → ARH projections unstimulated (yellow) or activated (blue). Results are shown as individual data points. **P < 0.01 between yellow vs. blue light in two-tailed paired *t* test (*N* = 6 mice per group). **O** The experimental paradigm to measure non-hunger-driven feeding. HFD intake during photostimulation and during the total 2.5-h period in mice with the 5-HT^DRN^ → ARH projections unstimulated (yellow) or activated (blue). Results are shown as individual data points (*N* = 6 mice per group). **P** Left: the experimental paradigm to measure real-time place preference in mice with the 5-HT^DRN^ → ARH projections unstimulated when in chamber 1 and activated when in chamber 2; right: the % time spent in each chamber in mice at hungry or satiated state with or without blue light stimulation coupled to chamber 2. Results are shown as mean ± SEM with individual data points. **P < 0.01 in two-way ANOVA analyses followed by Sidak post hoc test (*N* = 6 mice per group).

We next sought to clarify the target of 5-HT^DRN^ within the ARH. We focussed on anorexigenic pro-opiomelanocortin neurons and orexigenic agouti-related peptide neurons (POMC^ARH^ and AgRP^ARH^) because they are essential for feeding control [45–47]. We confirmed that TPH2-CreER does not induce Cre activity in the ARH, while POMC-CreER or AgRP-Cre does not induce Cre activity in the DRN (Fig. S2). We next stereotaxically injected Cre-dependent AAV expressing the optogenetic ChR2-EYFP into the DRN of the compound TPH2-CreER/POMC-CreER/Rosa26-LSL-tdTOMATO mice to express ChR2 specifically in 5-HT^DRN^ neurons and their fibers/terminals, which allows photostimulation. The same mice also received stereotaxic injections of Ad-iN/WED, an anterograde trans-synaptic viral tracer [42], into the DRN. Ad-iN/WED expressed GFP-tagged WGA (WGA-GFP) in a Cre-dependent manner and therefore 5-HT^DRN^ neurons and their downstream neurons were labeled by WGA-GFP. WGA-labeled somas in the ARH were identified as the immediate targets of 5-HT^DRN^ neurons. Note that these mice also express tdTOMATO in POMC^ARH^ neurons due to the combination of POMC-CreER and Rosa26-LSL-tdTOMATO alleles. Therefore, WGA(+)tdTOMATO(+) neurons within the ARH were identified as 5-HT^DRN^-innervated POMC^ARH^ neurons, which account for ~49% of POMC^ARH^ neurons [tdTOMATO(+), Fig. 1A–C].

Since WGA-GFP may also travel retrogradely [48], we adopted another approach to confirm the connectivity between 5-HT^DRN^ neurons and POMC^ARH^ neurons. CRACM was employed to examine ex vivo electrophysiological responses in ARH slices containing 5-HT^DRN^-innervated POMC^ARH^ [WGA(+)tdTOMATO(+)] neurons from TPH2-CreER/POMC-CreER/Rosa26-LSL-tdTOMATO mice. Under voltage-clamp, we detected blue light-evoked eEPSC only in 53% (10 out of 19) tested WGA(+)tdTOMATO(+) neurons (Fig. S3A–D). The eEPSCs were mediated by glutamatergic neurotransmission as they were blocked by glutamate receptor inhibitors (CNQX and D-AP5); 4-AP and TTX failed to affect the eEPSCs, confirming a monosynaptic nature (Fig. S3A–D). Interestingly, we also detected light-eIPSCs in a few (4 out of 21, 19%) WGA(+)tdTOMATO(+) neurons (Fig. S3E–H). The eIPSCs were mediated by GABA neurotransmission as they were blocked by GABA_A_ receptor inhibitors (bicuculline); 4-AP and TTX failed to affect the eIPSCs, confirming a monosynaptic nature (Fig. S3E–H). These results indicate that 5-HT^DRN^ neurons provide direct glutamatergic and/or GABAergic inputs to a portion of POMC^ARH^ neurons.

We then used a current-clamp mode to examine effects of blue light photostimulation on firing frequency and resting membrane potential in 5-HT^DRN^-innervated POMC^ARH^ [WGA(+)tdTOMATO(+)] neurons. Since AgRP^ARH^ neurons are known to provide local inhibitory GABAergic inputs to POMC^ARH^ neurons [49], we added 50 μM bicuculline, as well as 30 μM D-AP5 and 30 μM CNQX, in the bath solution to block the GABAergic and glutamatergic inputs in the current-clamp recordings. We found that blue light photostimulation on the 5-HT^DRN^ fibers/terminals in the ARH caused rapid increases in firing frequency and resting membrane potential in all tested WGA(+)tdTOMATO(+) neurons (Fig. 1D–F). We next examined the 5-HT receptor mediating this effect. These activations were completely abolished by a selective 5-HT_2C_R antagonist, 100 µM SB242084. These results indicate that photostimulation of 5-HT^DRN^ terminals activates POMC^ARH^ neurons via a 5-HT_2C_R-mediated mechanism. Notably, SB242084 also significantly reduced the baseline firing frequency and resting membrane potential (Fig. 1E, F), highlighting an excitatory effect of tonic 5-HT_2C_R actions on these anorexigenic POMC^ARH^ neurons.

We then used a similar approach in compound TPH2-CreER/AgRP-Cre/Rosa26-LSL-tdTOMATO mice to identify 5-HT^DRN^-innervated AgRP^ARH^ neurons, which account for ~32% of AgRP^ARH^ [tdTOMATO(+)] neurons (Fig. 1G–I). Using the same CRACM approach under the voltage-clamp protocol, we detected eEPSC in 65% (11 out of 17) tested WGA(+)tdTOMATO(+) neurons evoked by blue light pulses (Fig. S3I–L), and detected eIPSC in 20% (5 out of 25) tested WGA(+)tdTOMATO(+) neurons (Fig. S3M–P). Interestingly, under a current-clamp mode, we found that the blue light photostimulation caused rapid decreases in firing frequency and resting membrane potential in all tested WGA(+)tdTOMATO(+) neurons (Fig. 1J–L). Further, these inhibitions were completely abolished by a selective 5-HT_1B_R antagonist, 10 µM SB224289. These results indicate that activation of 5-HT^DRN^ neurons can inhibit AgRP^ARH^ neurons via a 5-HT_1B_R-mediated mechanism. Notably, addition of SB224289 also significantly enhanced the baseline resting membrane potential (Fig. 1J–L), highlighting an inhibitory effect of tonic 5-HT_1B_R actions on AgRP^ARH^ neurons.

Since 5-HT^DRN^ neurons activate anorexigenic POMC^ARH^ neurons and inhibit orexigenic AgRP^ARH^ neurons, we sought to examine the effects of 5-HT^DRN^ → ARH circuit on feeding control. To this end, we stereotaxically injected Cre-dependent AAV expressing ChR2-EYFP into the DRN of TPH2-CreER mice to express ChR2 specifically in 5-HT^DRN^ neurons and their fibers/terminals, and implanted the optic fiber to target the ARH (Fig. S3Q–R). After recovery, mice were first subjected to a hunger-driven feeding paradigm. Briefly, after overnight fasting, mice were provided with chow diet at 10 a.m. next morning, while photostimulation was applied for the first hour of refeeding period (Fig. 1N). Compared to mice receiving yellow light stimulation (as a control, 589 nm, 10 ms per pulse, 10 mW, 20 pulses per second), mice receiving blue light stimulation (473 nm, 10 ms per pulse, 10 mW, 20 pulses per second) displayed significantly reduced chow intake during the photostimulation period and also at the 2-h time point (Fig. 1N). We then examined dark-cycle feeding in chow-fed mice which is primarily driven by hunger, and found that photostimulation of the 5-HT^DRN^ → ARH circuit also significantly inhibited dark-cycle chow intake (Fig. S3S).

We then employed a non-hunger-driven feeding paradigm. Briefly, satiated mice were provided both chow and a HFD at 10 a.m. when mice normally do not eat (Fig. 1O). When provided with both chow and HFD, these satiated mice consumed primarily HFD but minimal chow (Fig. 1O and Fig. S3T), indicating that this HFD feeding is largely driven by the palatability of the HFD but rather by hunger. Importantly, there were no differences in chow or HFD intake between mice receiving yellow light or blue light stimulation (Fig. 1O and Fig. S3T). Next, we used a fasting paradigm that mice were first fasted overnight and then provided with both HFD and chow in the morning. In this setting, we found that photostimulation of the 5-HT^DRN^ → ARH circuit significantly reduced HFD intake in these hungry mice but had no effect on chow intake (Fig. S3U, V). These data suggest that the stimulation of the 5-HT^DRN^ → ARH circuit inhibits hunger-driven feeding, but has little effect on non-hunger-driven feeding behavior.

To examine the valence associated with 5-HT^DRN^ → ARH poststimulation, mice were assessed in a real-time place preference test. Briefly, mice were placed into a rectangular open field, which is comprised of two identical sides connected through an opening in the center (Fig. 1P). Without photostimulation, overnight fasted mice displayed no place preference (Fig. 1P). In the next trial, blue light pulses were paired with entry into the right compartment and fasted mice showed a preference for the blue light-paired area (Fig. 1P). However, in the satiated condition, mice did not show preference for the blue light-paired area (Fig. 1P). These data indicate that stimulation of the 5-HT^DRN^ → ARH circuit transmits a positive valence in hungry mice, but not in satiated mice, suggesting that the reduction in hunger-driven feeding is not due to the mice feeling unwell.

### 5-HT^DRN^ → VTA circuit inhibits non-hunger-driven feeding but not hunger-driven feeding

**Fig. 2:**
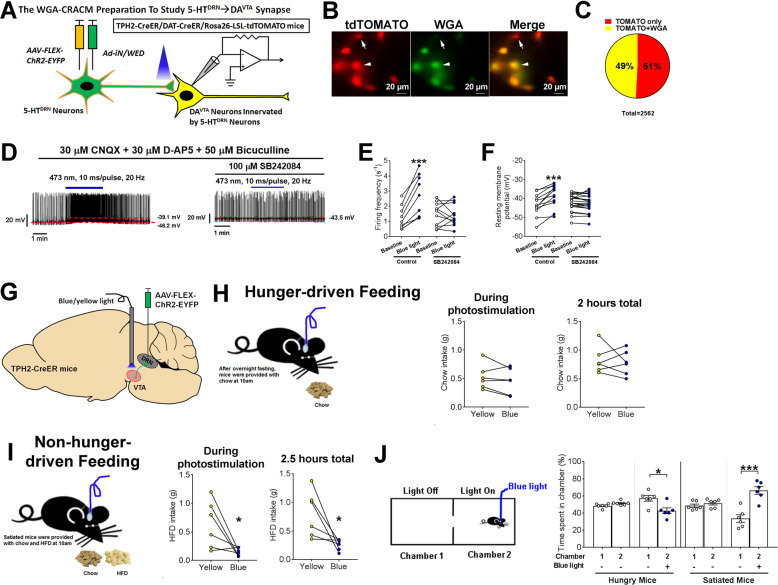
5-HT^DRN^ → VTA circuit inhibits non-hunger-driven feeding but not hunger-driven feeding. **A** Recordings in 5-HT^DRN^-innervated DA^VTA^ neurons [tdTOMATO(+)WGA(+)] in response to photostimulation of ChR2-labeled 5-HT^DRN^-originated fibers within the VTA. **B** Representative images showing VTA neurons labeled by tdTOMATO, WGA, or both. Scale bars = 20 µm. The arrow points to a single-labeled neuron and the arrowhead points to a double-labeled neuron. **C** The composition of tdTOMATO-labeled DA^VTA^ neurons that are tdTOMATO(+) only and tdTOMATO(+)WGA(+). A total of 2562 DA^VTA^ neurons were counted from five mice. **D** Representative action potential traces in response to blue light pulses (473 nm, 10 ms/pulse, 20 Hz) in the absence or the presence of 100 µM SB242084 in a bath solution containing 30 µM CNQX, 30 µM D-AP5, and 50 µM bicuculline. **E**, **F** Firing frequency (**E**) and resting membrane potential (**F**) at the baseline or after blue light stimulation in the absence or the presence of 100 µM SB242084. Results are shown as individual data points. ***P < 0.001 between blue light vs. baseline in two-way ANOVA analyses followed by Sidak post hoc test (*N* = 9–22 neurons from three mice per group). **G** Schematic experimental strategy using optogenetics to activate the 5-HT^DRN^ → VTA projections. **H** The experimental paradigm to measure hunger-driven feeding. Chow intake during photostimulation and during the total 2-h period in mice with the 5-HT^DRN^ → VTA projections unstimulated (yellow) or activated (blue). Results are shown as individual data points (*N* = 6 mice per group). **I** The experimental paradigm to measure non-hunger-driven feeding. HFD intake during photostimulation and during the total 2.5-h period in mice with the 5-HT^DRN^ → VTA projections unstimulated (yellow) or activated (blue). Results are shown as individual data points. *P <0.05 between yellow vs. blue light in paired *t* test (*N* = 6 mice per group). **J** Left: the experimental paradigm to measure real-time place preference in mice with the 5-HT^DRN^ → VTA projections unstimulated when in chamber 1 and activated when in chamber 2; right: the % time spent in each chamber in mice at hungry or satiated state with or without blue light stimulation coupled to chamber 2. Results are shown as mean ± SEM with individual data points. *P < 0.01 and ***P < 0.001 in two-way ANOVA analyses followed by Sidak post hoc test (*N* = 6 mice per group).

We next examined the effect of 5-HT^DRN^ innervation of neurochemically defined DA neurons within the VTA (DA^VTA^ neurons) because they are a key mediator of reward-related behaviors. We confirmed that TPH2-CreER does not induce Cre activity in the VTA, while the DAT-CreER does not induce Cre activity in the DRN (Fig. S2). Compound TPH2-CreER/DAT-CreER/Rosa26-LSL-tdTOMATO mice were used to identify 5-HT^DRN^-innervated DA^VTA^ neurons, which account for ~49% DA^VTA^ cells [tdTOMATO(+)] (Fig. 2A–C). Using a CRACM approach under the voltage-clamp protocol, we detected eEPSC in 84% (16 out of 19) tested WGA(+)tdTOMATO(+) neurons evoked by blue light pulses (Fig. S4A–D), and detected  eIPSC in 15% (4 out of 26) tested WGA(+)tdTOMATO(+) neurons (Fig. S4E–H).

Under a current-clamp mode, we found that blue light photostimulation caused rapid increases in firing frequency and resting membrane potential in all tested 5-HT^DRN^-innervated DA^VTA^ neurons [WGA(+)tdTOMATO(+)] (Fig. 2D–F). Further, these activations were completely abolished by a selective 5-HT_2C_R antagonist, 100 µM SB242084. These results indicate that stimulation of 5-HT^DRN^ neurons activates DA^VTA^ neurons via a 5-HT_2C_R-mediated mechanism. Notably, unlike responses seen in 5-HT^DRN^-innervated POMC^ARH^ neurons (Fig. 1E, F), the baseline firing frequency and resting membrane potential of these 5-HT^DRN^-innervated DA^VTA^ neurons were not significantly altered by SB242084 (Fig. 2E, F), indicating that 5-HT_2C_Rs have minor tonic actions on these DA^VTA^ neurons in a baseline condition.

We then examined the effects of 5-HT^DRN^ → VTA circuit on both hunger-driven and non-hunger-driven feeding behaviors, using TPH2-CreER mice with ChR2-EYFP expressed in 5-HT^DRN^ neurons and the optic fiber implanted to target the VTA (Fig. 2G and Fig. S4I). In the hunger-driven feeding paradigm, photostimulation of the 5-HT^DRN^ → VTA circuit by blue light pulses failed to significantly alter chow intake compared to the same mice receiving control yellow light stimulation (Fig. 2H). However, HFD intake during the non-hunger-driven feeding paradigm was significantly reduced by the photostimulation of the 5-HT^DRN^ → VTA circuit, while chow intake was not affected (Fig. 2I and Fig. S4J). These data indicate that the stimulation of the 5-HT^DRN^ → VTA circuit inhibits non-hunger-driven feeding, but has little effect on hunger-driven feeding. In the real-time place preference test, we observed that blue light photostimulation of the 5-HT^DRN^ → VTA circuit induced a mild negative valance in hungry mice, but strikingly, the same photostimulation transmitted a strong positive valence in satiated mice (Fig. 2J).

### 5-HT^DRN^ neurons increase activity during hunger-driven feeding and non-hunger-driven feeding

**Fig. 3:**
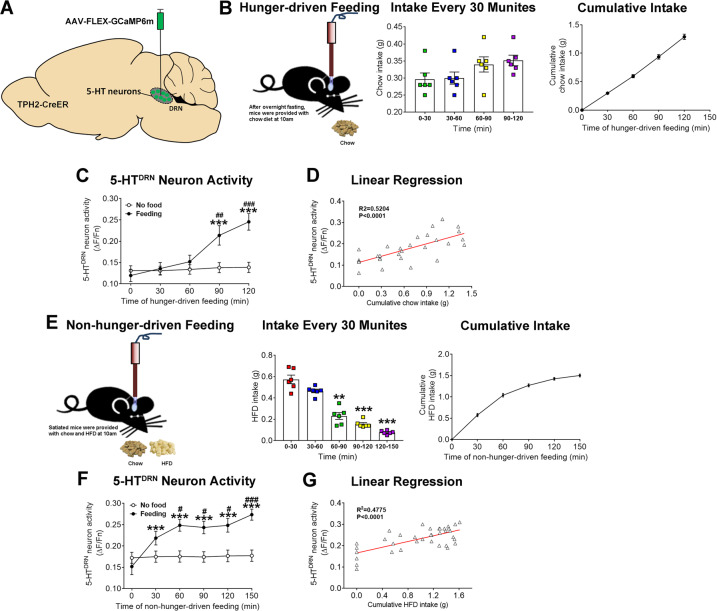
Dynamics of 5-HT^DRN^ neuron activity during hunger-driven feeding and non-hunger-driven feeding. **A** Schematic illustration to stereotaxically inject AAV-FLEX-GCaMP6m virus into the DRN of TPH2-CreER mice to express GCaMP6m in 5-HT^DRN^ neurons. **B** The schematic fiber photometry recordings of 5-HT^DRN^ neuron activity in mice during the course of hunger-driven feeding (left), the amount of chow intake during each 30-min bin within the 2-h refeeding (middle), and the cumulative chow intake (right). Results are shown as mean ± SEM with individual data points (*N* = 6 mice per group). **C** 5-HT^DRN^ neuron activity in mice during the course of 2-h hunger-driven feeding or in control hungry mice with no access to food. Results are shown as mean ± SEM (*N* = 5 or 6 mice per group). ***P < 0.001 vs. time 0; ##*P* < 0.01 and ###*P* < 0.001 vs. no food group in two-way ANOVA analyses followed by Sidak post hoc test. **D** The linear regression curve of cumulative chow intake and 5-HT^DRN^ neuron activity during hunger-driven feeding. Results are shown as individual data points with the linear regression curve plotted. **E** The schematic fiber photometry recordings of 5-HT^DRN^ neuron activity in mice during the course of non-hunger-driven feeding (left), the amount of HFD intake during each 30-min bin within 2.5-h feeding (middle), and the cumulative HFD intake (right). Results are shown as mean ± SEM with individual data points (*N* = 6 mice per group). **F** 5-HT^DRN^ neuron activity in mice during the course of 2.5-h non-hunger-driven feeding. Results are shown as mean ± SEM (*N* = 4 or 6 mice per group). ***P < 0.001 vs. time 0; #*P* < 0.05 and ###*P* < 0.001 vs. no food group in two-way ANOVA analyses followed by Sidak post hoc test. **G** The linear regression curve of cumulative HFD intake and 5-HT^DRN^ neuron activity during non-hunger-driven feeding. Results are shown as individual data points with the linear regression curve plotted.

We then examined the dynamic changes of 5-HT^DRN^ neuron activity during hunger-driven feeding and non-hunger-driven feeding in vivo in real time. To this end, we stereotaxically injected AAV-FLEX-GCaMP6m into the DRN of TPH2-CreER mice (with tamoxifen induction) to express the calcium sensor GCaMP6m selectively in 5-HT^DRN^ neurons and implanted the optic fiber to target the DRN, which allowed in vivo monitoring of 5-HT^DRN^ neuron activity in functional mice (Fig. 3A and Fig. S5A). Mice were subjected to the hunger-driven feeding paradigm. Overnight fasted mice ate 0.25–0.35 g of chow every 30 min, which resulted in a steady increase in cumulative chow intake during a 2-h refeeding period (Fig. 3B). Consistent with previous reports [40, 43], we observed rapid increases in 5-HT^DRN^ neuron activity that were tightly associated with onset of each eating bout and only lasted for a few seconds (Fig. S5B). Interestingly, we noted that 5-HT^DRN^ neuron activity also displayed a slow, but constant elevation during the 2-h feeding period (Fig. 3C). Linear regression analysis revealed a significant correlation between cumulative chow intake and 5-HT^DRN^ neuron activity (Fig. 3D).

We then subjected these mice to the non-hunger-driven feeding paradigm. Similarly, we observed transient elevations in 5-HT^DRN^ neuron activity associated with each eating bout (Fig. S5C). In addition, the 5-HT^DRN^ neuron activity displayed a slow elevation, which was significantly correlated with the cumulative HFD consumption (Fig. 3E–G). Together, these results indicate that during both hunger-driven and non-hunger-driven feeding, 5-HT^DRN^ neuron activity gradually escalates in the scale of minutes and hours, which is correlated with the amount of food consumed.

### GABA_A_ currents mediate dynamic changes in ARH-projecting 5-HT^DRN^ neuron activity during hunger-driven feeding

**Fig. 4:**
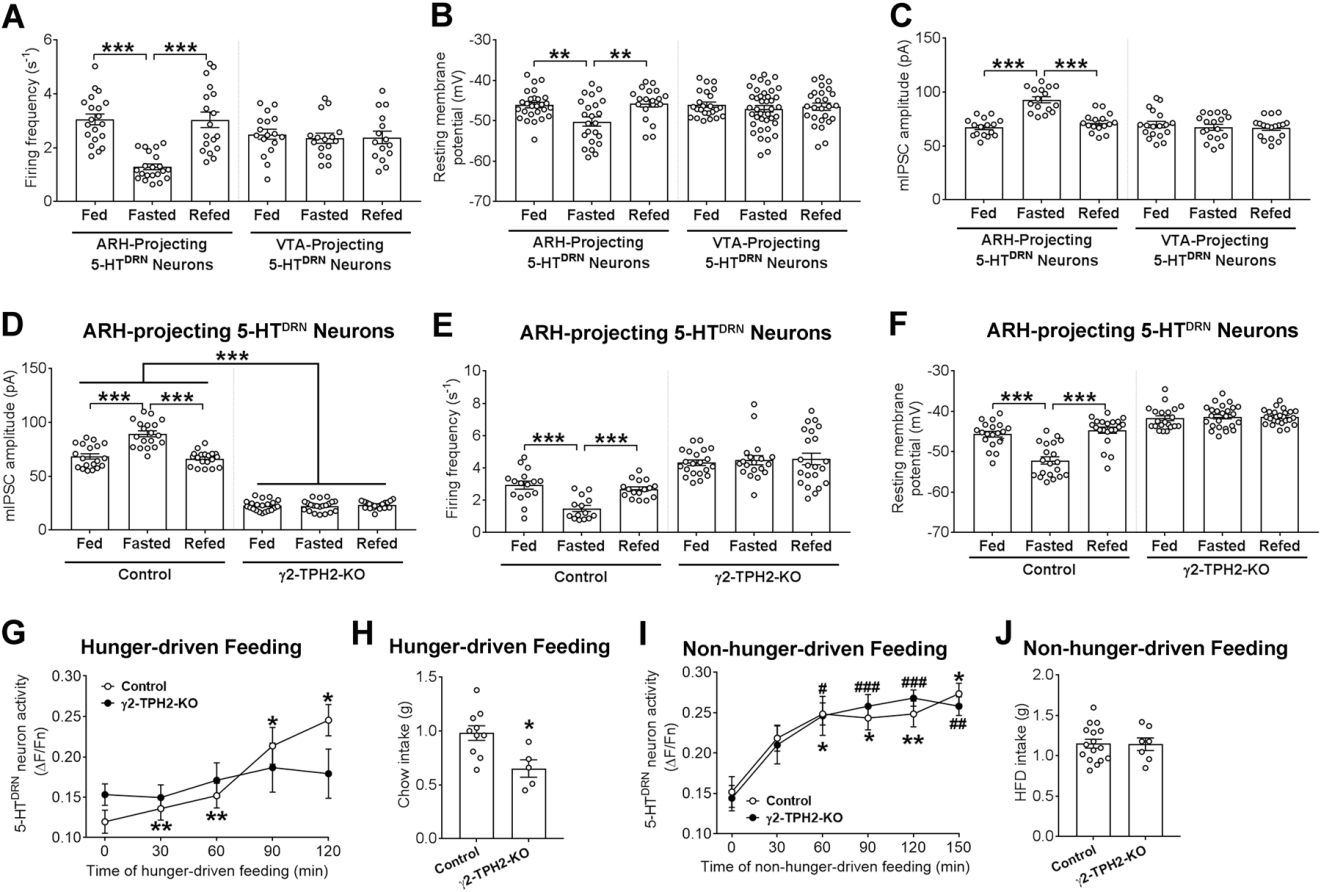
GABA_A_ currents mediate dynamic changes in ARH-projecting 5-HT^DRN^ neuron activity during hunger-driven feeding. **A**, **B** Firing frequency (**A**) and resting membrane potential (**B**) in ARH-projecting or VTA-projecting 5-HT^DRN^ neurons in mice fed ad libitum, fasted for overnight, or fasted for overnight followed by 2-h refeeding. Results are shown as mean ± SEM with individual data points. **P < 0.01 and ***P <0.001 in one-way ANOVA analyses followed by Sidak post hoc test (*N* = 15–27 neurons from three mice per group). **C** Amplitude of mIPSC in ARH-projecting or VTA-projecting 5-HT^DRN^ neurons in mice fed ad libitum, fasted for overnight, or fasted for overnight followed by 2-h refeeding. Results are shown as mean ± SEM with individual data points. ***P < 0.001 in one-way ANOVA analyses followed by Sidak post hoc test (*N* = 16–17 neurons from three mice per group). **D**–**F** Amplitude of mIPSC (**D**), firing frequency (**E**), and resting membrane potential (**F**) in ARH-projecting 5-HT^DRN^ neurons in control or γ2-TPH2-KO mice fed ad libitum, fasted for overnight, or fasted for overnight followed by 2-h refeeding. Results are shown as mean ± SEM with individual data points. ***P < 0.001 in two-way ANOVA analyses followed by Sidak post hoc test (*N* = 14–25 neurons from three mice per group). **G** Averaged 5-HT^DRN^ neuron activity in control or γ2-TPH2-KO mice during the course of 2-h hunger-driven feeding. Note that the data from control mice are the duplicate of data described in Fig. 3C. Results are shown as mean ± SEM. *P < 0.05 and **P < 0.01 vs. time 0 in control mice in two-way ANOVA analyses followed by Sidak post hoc test (*N* = 5 or 6 mice per group). **H** The amount of chow intake during 2-h hunger-driven feeding in control or γ2-TPH2-KO mice. Results are shown as mean ± SEM with individual data points. *P < 0.05 in *t* test (*N* = 5 or 10 mice per group). **I** Averaged 5-HT^DRN^ neuron activity in control or γ2-TPH2-KO mice during the course of 2.5-h non-hunger-driven feeding. Note that the data from control mice are the duplicate of data described in Fig. 3F. Results are shown as mean ± SEM. *P < 0.05 and **P < 0.01 vs. time 0 in control mice; #*P* < 0.05, ##*P* < 0.01, and ###*P* < 0.001 vs. time 0 in γ2-TPH2-KO mice in two-way ANOVA analyses followed by Sidak post hoc test (*N* = 5 or 6 mice per group). **J** The amount of HFD intake during 2.5-h non-hunger-driven feeding in control or γ2-TPH2-KO mice. Results are shown as mean ± SEM with individual data points (*N* = 7 or 16 mice per group).

We next explored whether the activity of 5-HT^DRN^ neurons projecting to the ARH and/or VTA changes in association with hunger-driven feeding. To this end, TPH2-CreER/Rosa26-LSL-tdTOMATO mice were stereotaxically injected with green and purple RetroBeads into the ARH and VTA, respectively (Fig. S6A). Brain slices containing the DRN were freshly prepared from mice fed ad libitum, overnight fasted, or overnight fasted followed by 2-h refeeding. Electrophysiological recordings were performed in tdTOMATO-labeled 5-HT^DRN^ neurons projecting to the ARH (co-labeled by green beads) or in tdTOMATO-labeled 5-HT^DRN^ neurons projecting to the VTA (co-labeled by purple beads, Fig. S6A). The firing frequency and resting membrane potential of ARH-projecting 5-HT^DRN^ neurons in fasted mice were significantly reduced compared to fed mice, and 2-h refeeding increased firing frequency and resting membrane potential to the level of fed mice (Fig. 4A, B). On the other hand, firing frequency and resting membrane potential of VTA-projecting 5-HT^DRN^ neurons were not affected by these conditions (Fig. 4A, B).

To further explore the mechanisms for the dynamic 5-HT^DRN^ neuron activity during the hunger-driven feeding, we examined the mEPSC and the SK currents in ARH- and VTA-projecting 5-HT^DRN^ neurons, and did not observe any significant changes in these currents in the different feeding conditions (Fig. S6B–D). However, the amplitude of miniature IPSC (mIPSC) in ARH-projecting 5-HT^DRN^ neurons was significantly enhanced by fasting compared to fed mice, and 2-h refeeding largely restored mIPSC amplitude to the level of fed mice. mIPSC amplitude in VTA-projecting 5-HT^DRN^ neurons was not affected (Fig. 4C). No differences were observed in the frequency of mIPSC by feeding conditions (Fig. S6E). Given the inhibitory nature of mIPSC, we hypothesized that GABA-mediated mIPSC contributes to the dynamic ARH-projecting 5-HT^DRN^ neuron activity during the course of hunger-driven feeding.

To examine this, we generated γ2-TPH2-KO mice which lack the pore-forming γ2 subunit of GABA_A_ receptor [31, 50, 51] specifically in 5-HT neurons and their control littermates (Fig. S6F). We found that mIPSC amplitude, but not frequency, in ARH-projecting 5-HT^DRN^ neurons from γ2-TPH2-KO mice was significantly smaller than control mice, and the dynamic pattern of mIPSC we observed in control mice during the hunger-driven feeding was abolished in γ2-TPH2-KO mice (Fig. 4D and Fig. S6G). Importantly, the firing frequency and resting membrane potential of ARH-projecting 5-HT^DRN^ neurons from γ2-TPH2-KO mice did not display the dynamic alterations during the course of hunger-driven feeding, as seen in those from control mice (Fig. 4E, F). Consistently, fiber photometry demonstrated that 5-HT^DRN^ neuron activity in γ2-TPH2-KO mice was not gradually regulated during the course of hunger-driven feeding (Fig. 4G), although the transient elevations associated with onset of eating bouts were still observed (Fig. S6H) similarly as those in control mice (Fig. S5B). Importantly, in the hunger-driven feeding paradigm, the amount of chow intake during the 2-h refeeding period was significantly reduced compared to control mice (Fig. 4H). However, during non-hunger-driven feeding, 5-HT^DRN^ neuron activity in γ2-TPH2-KO mice displayed similar elevations and HFD and chow intake were comparable to control mice (Fig. 4I, J, S6I). Together, these results indicate that GABA_A_ currents mediate dynamic changes in ARH-projecting 5-HT^DRN^ neuron activity during hunger-driven feeding.

### SK currents mediate dynamic changes in VTA-projecting 5-HT^DRN^ neuron activity during non-hunger-driven feeding

**Fig. 5:**
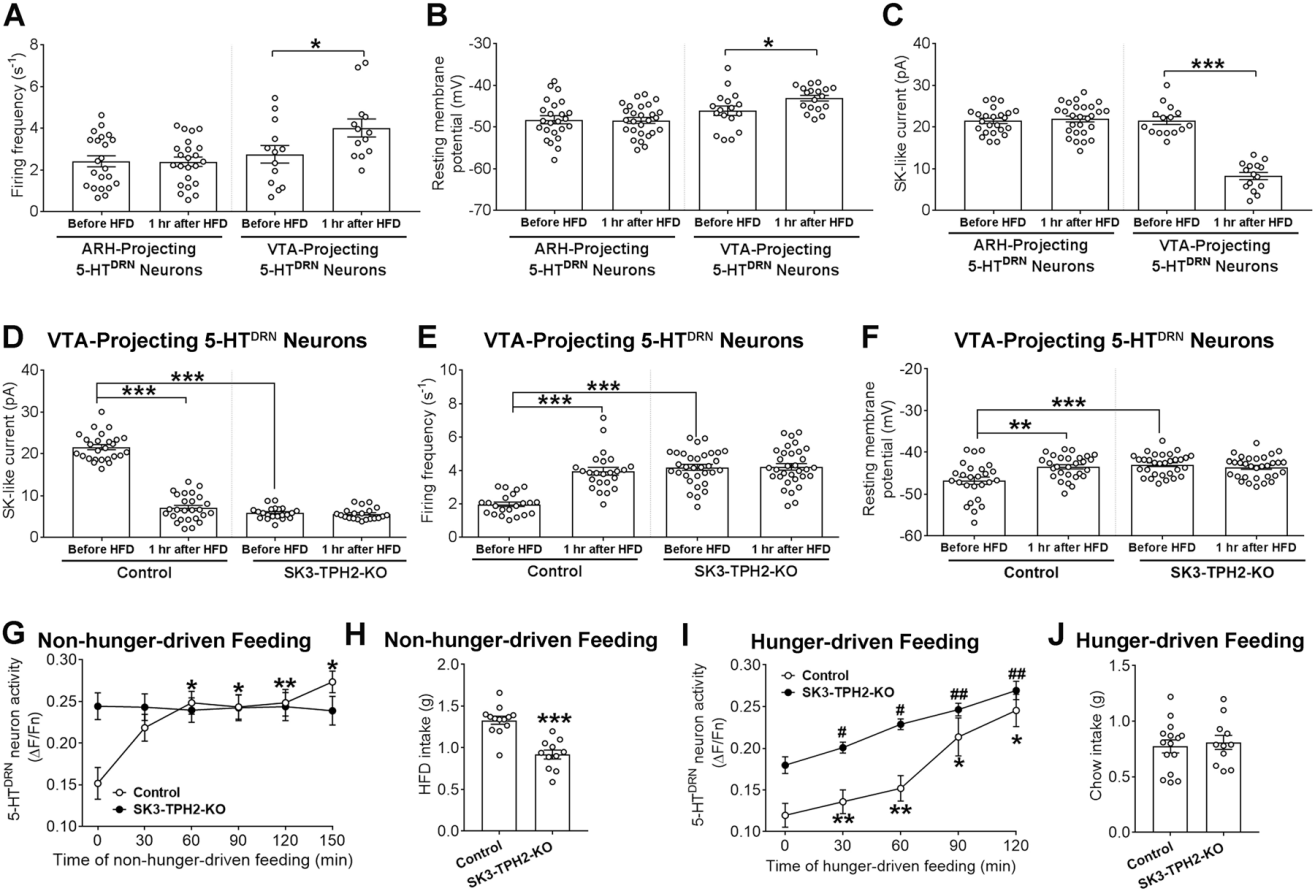
SK currents mediate dynamic changes in VTA-projecting 5-HT^DRN^ neuron activity during non-hunger-driven feeding. **A**, **B** Firing frequency (**A**) and resting membrane potential (**B**) in ARH-projecting or VTA-projecting 5-HT^DRN^ neurons in mice before and 1-h after non-hunger-driven feeding. Results are shown as mean ± SEM with individual data points. *P < 0.05 in *t* test (*N* = 13–27 neurons from three mice per group). **C** Amplitude of SK-like currents in ARH-projecting or VTA-projecting 5-HT^DRN^ neurons in mice before and 1-h after non-hunger-driven feeding. Results are shown as mean ± SEM with individual data points. ***P < 0.001 in *t* test (*N* = 14–27 neurons from three mice per group). **D**–**F** Amplitude of SK-like currents (**D**), firing frequency (**E**), and resting membrane potential (**F**) in VTA-projecting 5-HT^DRN^ neurons in control or SK3-TPH2-KO mice before and 1-h after non-hunger-driven feeding. Results are shown as mean ± SEM with individual data points. **P < 0.01 and ***P < 0.001 in two-way ANOVA analyses followed by Sidak post hoc test (*N* = 21–32 neurons from three mice per group). **G** Averaged 5-HT^DRN^ neuron activity in control or SK3-TPH2-KO mice during the course of 2.5-h non-hunger-driven feeding. Note that the data from control mice are the duplicate of data described in Fig. 3F. Results are shown as mean ± SEM. *P < 0.05 and **P < 0.01 vs. time 0 in control mice in two-way ANOVA analyses followed by Sidak post hoc test (*N* = 5 or 7 mice per group). **H** The amount of HFD intake during 2.5-h non-hunger-driven feeding in control or SK3-TPH2-KO mice. Results are shown as mean ± SEM with individual data points. ***P < 0.001 in *t* test (*N* = 11 or 13 mice per group). **I** Averaged 5-HT^DRN^ neuron activity in control or SK3-TPH2-KO mice during the course of 2-h hunger-driven feeding. Note that the data from control mice are the duplicate of data described in Fig. 3C. Results are shown as mean ± SEM. *P < 0.05 and **P < 0.01 vs. time 0 in control mice; #*P* < 0.05 and ##*P* < 0.01 vs. time 0 in SK3-TPH2-KO mice in two-way ANOVA analyses followed by Sidak post hoc test (*N* = 5 or 6 mice per group). **J** The amount of chow intake during 2-h hunger-driven feeding in control or SK3-TPH2-KO mice. Results are shown as mean ± SEM with individual data points (*N* = 11 or 15 mice per group).

We also examined the ex vivo dynamic firing activities of ARH- and/or VTA-projecting 5-HT^DRN^ neurons during the course of non-hunger-driven feeding (Fig. S6A). Firing frequency and resting membrane potential of VTA-projecting 5-HT^DRN^ neurons were significantly increased 1 h after non-hunger-driven feeding compared to the baseline levels, whereas the activity of ARH-projecting 5-HT^DRN^ neurons did not change (Fig. 5A, B). We did not detect any significant changes in mIPSC or mEPSC in these neurons (Fig. S7A–D). Interestingly, SK currents in VTA-projecting 5-HT^DRN^ neurons, but not in ARH-projecting neurons, were significantly reduced 1 h after non-hunger-driven feeding (Fig. 5C).

To examine the physiological role of SK currents in 5-HT^DRN^ neurons in feeding control, we generated control littermates and SK3-TPH2-KO mice with 5-HT-specific deletion of SK3 (Fig. S7E), the most abundant SK isoform in the DRN [52]. SK currents in VTA-projecting 5-HT^DRN^ neurons from SK3-TPH2-KO mice were significantly smaller than control mice, and the dynamic pattern of SK currents we observed in control mice during non-hunger-driven feeding was abolished in SK3-TPH2-KO mice (Fig. 5D). Importantly, the firing frequency and resting membrane potential of VTA-projecting 5-HT^DRN^ neurons from SK3-TPH2-KO mice were significantly elevated, and they did not display the dynamic alterations during the course of non-hunger-driven feeding as seen in those from control mice (Fig. 5E, F). Consistently, we used fiber photometry to demonstrate that unlike control mice, the 5-HT^DRN^ neuron activity in SK3-TPH2-KO mice did not alter during the course of non-hunger-driven feeding (Fig. 5G), although the transient elevations associated with onset of eating bouts were still observed (Fig. S7F). Importantly, the amount of HFD intake, but not chow intake, during the non-hunger-driven feeding paradigm was significantly reduced compared to control mice (Fig. 5H and Fig. S7G). Interestingly, during hunger-driven feeding, the 5-HT^DRN^ neuron activity in SK3-TPH2-KO mice, although starting from a higher baseline level, displayed similar elevations as in control mice, and their refeeding behavior was comparable (Fig. 5I, J). Together, these results indicate that SK currents mediate dynamic changes in VTA-projecting 5-HT^DRN^ neuron activity during non-hunger-driven feeding. We then subjected control and SK3-TPH2-KO mice to the open-field test, the light–dark test, the elevated plus maze test, and forced swim test, and found comparable locomotor activity, anxiety-like, and depression-like behaviors in these mice (Fig. S8). Thus, we suggest that the reduced non-hunger-driven feeding in these mice was not secondary to changes in animals’ locomotion and mood. Finally, we compared SK3 protein levels in the DRN before and after mice conducted non-hunger-driven feeding, and found that 2.5-h non-hunger-driven feeding significantly reduced SK3 levels in the DRN (Fig. S9).

## Discussions

The primary cause of the global obesity epidemic is the overconsumption of food, which is driven by hunger-dependent and -independent mechanisms. Here, we aimed to define specific brain circuits modulating hunger-driven and non-hunger-driven feeding behavior with the objective of informing the rational design of precision medicines for the treatment of obesity. We employed a commonly used paradigm [37, 53, 54] to measure hunger-driven feeding as a rapid chow refeeding behavior induced by overnight fasting. Non-hunger-driven feeding was assessed as HFD intake in satiated mice presented with a free choice of HFD and chow diet; importantly, we only measured HFD intake for 2.5 h during the morning, instead of a longer period (e.g., 12 and 24 h) or during the late afternoon/evening, which should minimize food intake triggered by hunger or circadian cues. Of course, one limitation is that we did not measure food intake in longer periods and may have missed potential rebound in feeding. Nevertheless, using these feeding paradigms, we provide the insight into the in vivo activity of 5-HT^DRN^ neurons in response to different types of foods and reveal that 5-HT^DRN^ neurons progressively increase their activity during food consumption. Further, we report that activation of the 5-HT^DRN^ → ARH projections efficiently decreased hunger-driven feeding, but had no effect on non-hunger-driven feeding, whereas activation of the 5-HT^DRN^ → VTA projections robustly inhibited non-hunger-driven feeding, but had no effect on hunger-driven feeding.

The ARH was first demonstrated to be a necessary homeostatic feeding center through lesion studies in last century, and the past few decades have revealed the importance of POMC^ARH^ and AgRP^ARH^ neurons in mediating this effect [55, 56]. We and others have reported that 5-HT, like other hormones and neurotransmitters, regulates the activity of POMC^ARH^ and AgRP^ARH^ neurons [57–59]. However, the source of 5-HT sufficient to modulate the POMC^ARH^ and AgRP^ARH^ activity, and feeding behavior has not been fully clarified. Here, we report that 5-HT^DRN^ neurons innervate around half of anorexigenic POMC^ARH^ cells via 5-HT_2C_Rs and a subset of orexigenic AgRP^ARH^ neurons via 5-HT_1B_Rs. We report that all 5-HT^DRN^-innervated POMC^ARH^ neurons were activated by 5-HT_2C_R-mediated 5-HTergic inputs. Notably, since these current-clamp recordings were performed in the presence of GABA and glutamate receptor inhibitors to block the majority of fast synaptic inputs, we suggest that the observed rapid activations in tested neurons likely reflect responses to direct synaptic inputs from 5-HT^DRN^ neurons. It is striking that in the presence of GABA and glutamate receptor inhibitors, the 5-HT_2C_R antagonist (SB242084) completely abolished the spontaneous firing of 5-HT^DRN^-innervated POMC^ARH^ neurons. Thus, we suggest that the 5-HT inputs (acting through 5-HT_2C_Rs) and glutamatergic inputs provide the majority of excitatory drive to this subset of POMC^ARH^ neurons. These findings are consistent with earlier reports that 5-HT_2C_R agonists activate a portion of POMC^ARH^ neurons [14, 58]. Further, deletion of 5-HT_2C_Rs from POMC neurons leads to hyperphagia and obesity in mice [26] and 5-HT_2C_Rs expressed by POMC neurons partly mediates anorexigenic effects of 5-HT analogs during hunger-driven feeding [60]. We also observed that all 5-HT^DRN^-innervated AgRP^ARH^ neurons were inhibited by 5-HT_1B_R-mediated 5-HTergic inputs. These results are consistent with previous reports that 5-HT_1B_Rs inhibit AgRP^ARH^ neurons and 5-HT_1B_R agonists reduce hunger-driven feeding [57]. Together, these observations indicate that a subset of 5-HT^DRN^ neurons project to the ARH, where they activate POMC^ARH^ neurons via 5-HT_2C_Rs and inhibit AgRP^ARH^ neurons via 5-HT_1B_Rs to reduce hunger-driven feeding. Such findings suggest that in addition to the 5-HT_2C_R, the 5-HT_1B_R may be another suitable target for the treatment of obesity. Indeed, co-administration of 5-HT_2C_R and 5-HT_1B_R agonists in animals produces a greater reduction in food intake compared to single agonist treatment [61].

A separate population of 5-HT^DRN^ neurons innervate a subset of DA^VTA^ neurons. DA^VTA^ neurons are instrumental in modulating motivation and reward-related behaviors. We report that 5-HT^DRN^ influences the activity of DA^VTA^ neurons, and this effect is mediated via the 5-HT_2C_Rs. In line with this finding, we previously reported that lorcaserin activates DA^VTA^ neurons, effects that are blocked by deletion of 5-HT_2C_Rs in DA neurons [15]. Further, deletion of 5-HT_2C_Rs in DA neurons blocks effects of lorcaserin to inhibit binge-like eating (a feeding behavior largely driven by hedonic value of high palatable food), but not hunger-driven feeding [15]. Together, these results indicate that a subset of 5-HT^DRN^ neurons project to and activate DA^VTA^ neurons via 5-HT_2C_R-mediated mechanisms, which inhibits non-hunger-driven feeding. In addition to the VTA and ARH, 5-HT_2C_Rs are also expressed in other brain regions, e.g., the lateral hypothalamic area and the ventral hippocampus [62], both of which are involved in feeding control [63–65]. The potential role of 5-HT_2C_Rs in these brain regions warrant future investigations.

Notably, a small portion of 5-HT^DRN^ neurons do have collateral projections to both the ARH and VTA, which may confound the circuit-specific stimulation. Nevertheless, given the drastic differences in the phenotypic outcome associated with the photostimulation of these two projections, we suggest that these collaterals likely play a minor role in our experimental settings. Thus, our findings are in line with the notion that neural circuits regulating hunger-driven feeding vs. non-hunger-driven feeding are segregated. Consistently, while animals without AgRP^ARH^ neurons can appropriately consume high palatable food [66], these neurons are dispensable for normal feeding responses induced by hunger signals, e.g., ghrelin [66] and asprosin [67]. However, it has to be pointed out that these two feeding-regulatory systems can interplay with each other. For instance, ghrelin can enhance the responses of DA^VTA^ neurons to hedonic cues [68], whereas chronic HFD feeding reduces the responses of AgRP^ARH^ neurons to ghrelin [35]. Indeed, DA^VTA^ neurons are reported to receive indirect inputs from AgRP^ARH^ neurons [35] and POMC^ARH^ neurons [33], which may provide anatomical basis for the crosstalk between the homeostatic and the hedonic circuitry.

In addition to 5-HT-mediated neurotransmissions, we also noted that 5-HT^DRN^ neurons provide glutamatergic or GABAergic inputs to a portion (but not all) of innervated neurons. This is consistent with recent findings that 5-HT^DRN^ neurons are partially overlapping with glutamatergic or GABAergic neurons [43, 44]. Notably, hunger-driven feeding can be inhibited by selective stimulation of glutamatergic neurons in the DRN or by inhibition of GABAergic neurons in the DRN [69]. We found that in the presence of glutamate and GABA receptor inhibitors, 5-HT^DRN^ neurons can provide 5-HTergic inputs to all tested ARH neurons in the in vitro preparation. However, we cannot fully rule out the in vivo contribution of glutamatergic and/or GABAergic outputs from 5-HT^DRN^ neurons to the regulation of hunger-driven feeding, as well as non-hunger-driven feeding. Future studies are warranted to further dissect out the contributions of 5-HT signaling, vs. glutamate or GABA co-released by 5-HT^DRN^ neurons, to the regulation of feeding behavior.

It has been reported that 5-HT^DRN^ neurons can be transiently (on a time scale of seconds) activated by various stimuli, e.g., sex, social interaction, sucrose, or food pellet intake [40, 43]. Consistently, we observed transient activations of 5-HT^DRN^ neurons associated with onset of eating bouts (either chow or HFD). However, we found that these eating bout-associated transient elevations in 5-HT^DRN^ neurons persisted in γ2-TPH2-KO and SK3-TPH2-KO mice. Given these mutant mouse models displayed altered hunger-driven or non-hunger-driven feeding, we suggest that the transient elevations in 5-HT^DRN^ neurons do not have regulatory effects on feeding behavior per se, although they may encode certain aspects of eating, e.g., initiation of an eating bout or detection of food. On the other hand, we found that 5-HT^DRN^ neurons also chronically increased their activities during the course of feeding on a time scale of minutes to hours. Considering that activation of 5-HT^DRN^ neurons inhibits feeding, we suggest that 5-HT^DRN^ neurons function as a key component of a negative feedback loop. Thus, feeding, either driven by hunger or by hunger-independent cues, gradually activates 5-HT^DRN^ neurons, and this slowly elevated 5-HT^DRN^ neuron activity then terminates the meal to prevent overeating. Notably, meal termination can be regulated by other neural populations, including calcitonin gene-related peptide-expressing neurons in the external lateral parabrachial nucleus [70] and catecholamine neurons in the nucleus of solitary tract [71]. It remains unclear whether these neural nodes, including 5-HT^DRN^ neurons, function in a parallel fashion, or within an interconnected network to regulate meal termination.

The slow dynamic changes in 5-HT^DRN^ neurons are not associated with presynaptic alternations (e.g., changes in frequencies of mEPSC and mIPSC), which could happen in seconds. Rather, distinct postsynaptic mechanisms are involved in the regulations of 5-HT^DRN^ neuron activity during hunger-driven vs. non-hunger-driven feeding. On one hand, hunger-driven feeding increased ARH-projecting 5-HT^DRN^ neuron activity by reducing the amplitude of mIPSC. On the other hand, non-hunger-driven feeding activated VTA-projecting 5-HT^DRN^ neurons by reducing a SK outward potassium current. It is worth noting that comparable mIPSCs were detected in ARH- and VTA-projecting 5-HT^DRN^ neurons, although only the mIPSC in the former subset was regulated by hunger-driven feeding. Similarly, comparable SK currents were detected in ARH- and VTA-projecting 5-HT^DRN^ neurons, although only the SK current in the latter subset was regulated by non-hunger-driven feeding. Thus, while both these currents exist in the two subsets of 5-HT^DRN^ neurons, their responses to hunger-driven feeding and non-hunger-driven feeding may involve different regulatory mechanisms which remain unclear. Since 5-HT^DRN^ neuron activity was correlated with the amount of chow or HFD intake, we speculate that the consumed nutrients, calories, and/or the nutritional state of the animal may contribute to the slow dynamic changes in 5-HT^DRN^ neurons, although such possibilities need to be further validated. In addition, our observations that non-hunger-driven feeding can reduce SK3 protein levels in the DRN suggest that carbohydrates and/or lipids in the diets may regulate levels of ion channels, and therefore influence neural activity.

It is worth mentioning that the role of 5-HT^DRN^ neurons in the reward-related behaviors are complex. Correia et al. reported no place preference or aversion associated with repetitive place-contingent pairing of optogenetic activation of 5-HT^DRN^ neurons [72]. However, multiple groups demonstrated that optogenetic activation of 5-HT^DRN^ neurons or the 5-HT^DRN^ → VTA projections increase place preference and other reward-related behaviors [73, 74]. Consistent with the latter, here we found that activation of the 5-HT^DRN^ → VTA projections increased place preference in satiated mice, which was, however, totally reversed in hungry mice. On the other hand, activation of the 5-HT^DRN^ → ARH projections increased place preference only in hungry mice, but not in satiated mice. Thus, our results add to this complexity and suggest that 5-HT^DRN^ neurons play different roles in regulating place preference depending on the different downstream neural circuits and on the nutritional states of animals. Similarly, a circuit from the paraventricular hypothalamus to the lateral parabrachial nucleus has been reported to increase place preference only in hungry mice, an effect that is completely blocked in satiated mice [75].

Owing to the diverse etiologies of obesity, it has been difficult to treat with medication and is therefore ideally suited to a precision medication approach. Here, we investigated specific brain regions underpinning different types of feeding behaviors. We report that hunger-driven and non-hunger-driven feeding can gradually activate 5-HT^DRN^ neurons, via either inhibiting responsiveness to inhibitory GABAergic inputs or by reducing a potassium outward current. Activated 5-HT^DRN^ neurons in turn inhibit hunger-driven and non-hunger-driven feeding through their projections to the ARH and VTA, respectively. These 5-HT^DRN^-originated neural circuits constitute a negative feedback loop to trigger the end of a meal. These findings suggest that individuals with increased hunger could benefit from a medication targeting the 5-HT^DRN^ → ARH circuit, individuals who overeat in the absence of hunger could benefit from a medication targeting the 5-HT^DRN^ → VTA circuit.

## Supplementary information

Supplementary methods and figures
