# Supplementary material for: 5-HT recruits distinct neurocircuits to inhibit hunger-driven and non-hunger-driven feeding
Source: Mol Psychiatry. 2021 Jul 21;26(12):7211–24. doi: 10.1038/s41380-021-01220-z (PMC8776930; doi:10.1038/s41380-021-01220-z)
Supplement: Supplementary file 1 — Supplementary methods and figures [file 41380_2021_1220_MOESM1_ESM.docx]

**Supplemental Methods and Materials**

**Anxiety Tests (Open Field Test, Light-Dark Test and Elevated Plus Maze)**

To determine if SK3-TPH2-KO mice have anxiety-related phenotypes, an independent cohort of SK3-TPH2-KO and control littermates were subjected to the open field tests, the light-dark tests, and the elevated plus maze tests, as we did before [^1^](#_ENREF_1)^,^ [^2^](#_ENREF_2). Note that all the behavioral tests were performed at around 9-10 am during the light cycles. Briefly, the open-field test was performed in a clear Plexiglas open-field arena (40 cm X 40 cm X30 cm). Mice were first placed into the center of the arena and allowed to explore for 30 min. Overhead lighting and white noise were present to provide ~800 lux illumination and ~55 dB sound inside the arena. Data were collected in 2 min intervals by a computer-operated Digiscan optical animal activity system (RXYZCM, Accuscan Electronics). For the current study, the data over the total 30-min test session were analyzed. Total distance traveled and center time (the time traveled in the center of the arena) were recorded. Center time can be used as an index of anxiety-related responses. The number of boli was also measured at the end of testing.

On a different day, these mice were subjected to the light-dark tests. The light-dark test consisted of a polypropylene chamber (44 × 21 × 21 cm) unequally divided into a larger, brightly illuminated open compartment (clear polypropylene) and a smaller, dark compartment (in dark polypropylene), connected by a small opening. Mice were placed in the illuminated chamber and allowed to move freely between the two chambers for 10 min. The latency to enter the light and dark chambers, the time spent in the chambers, the total number of transitions, and the ratio of time in light/dark chamber were measured using the VersaMax Animal Activity Monitoring System (AccuScan Instruments, Inc., OH) and analyzed. Transfer of all four paws of an experimental animal from one chamber to the other was considered as one transition event.

On a different day, these mice were subjected to the elevated plus maze (EPM). The EPM was constructed of plexiglas with two open arms (30 × 5 cm) and two enclosed black arms (30 × 5 × 15 cm) at an elevation of 50 cm above the floor. The arms of the maze form a cross with the two open arms facing each other. The maze was cleaned with 70% ethanol solution after each session and allowed to dry between the sessions. Anxiety-like behavior was measured by placing the mice in the center of the junction of the arms of the maze facing an open arm and the behavior analyzed for 10 min. The numbers of entries into the open and closed arms, the time spent exploring the open and closed arms, and distance traveled were recorded and analyzed using the ANY-maze software (Stoelting Co., Wood Dale, IL). The changes in anxiety-like behavior were calculated by dividing the number of entries into the open arms by dividing the amount of time spent in the open arms by the amount of time spent in all four arms (open time ratio) and by the total number of entries into all four arms (open entry ratio). The time spent in the center platform not exploring any of the arms was not included in these calculations.

**Forced Swim Test**

To determine if SK3-TPH2-KO mice have depression-related phenotypes, SK3-TPH2-KO and control littermates were subjected to the forced swim test as we did before [^1^](#_ENREF_1)^,^ [^3^](#_ENREF_3). Briefly, the mice were individually placed into a glass cylinder (25 cm tall X 10 cm diameter) containing 8 cm of water, maintained at 23–25°C for 6 minutes. The mouse movement was digitally recorded from the side using a camcorder. Water in the cylinder was replaced after each recording. The starting and total time of immobility for each mouse were recorded. Immobility is defined as the absence of active, floating with only minimal movements needed to keep head above water. The immobility was scored every 30 seconds for the last 4 minutes. During each 30 seconds, 1 immobility score was recorded if the immobility time was more than 10 seconds. Data from mice that had difficulty keeping heads above water were excluded from analyses. All the videos were blindly rated by a different experimenter.

**Western Blot**

Male C57Bl6j mice (12 weeks) were first trained in the non-hunger feeding paradigm as described above. On the day of non-hunger feeding, some mice were euthanized immediately before the non-hunger feeding started, and some mice were euthanized at 2.5 hours after the non-hunger feeding started. Briefly, mice were deeply anesthetized with isoflurane (4–5%), and the brains were rapidly dissected and frozen on dry ice. The DRN region was microdissected by brain punch (57402, Hedebio , Beijing) and total protein was also extracted. The protein concentration was assayed using BCA protein assays (23225, Thermo Fisher Scientific). After separation on 10% SDS-PAGE gels, the proteins were transferred to polyvinylidene fluoride membranes. Membranes were then subjected to immunoblotting with primary antibodies against SK3 (1:2000, APC-025, Alomone Labs) and β-actin (1:5000, bs-0061R, Bioss, China), followed by goat-anti-rabbit HRP conjugated secondary antibody (1:50000, bs-0295G, Bioss, China) for 1.5 hours at room temperature. Protein expression was measured using a Fluorescence Imaging System (ProteinSimple, Santa Clara, CA, USA) and qualified by Image J software.

**KEY RESOURCES TABLE**

| **REAGENT or RESOURCE** | **SOURCE** | **IDENTIFIER** |
| --- | --- | --- |
| **Antibodies** | | |
| Rabbit anti-5-HT antibody | ImmunoStar | 20080 |
| Goat anti-5-HT antibody | ImmunoStar | 20079 |
| Goat anti-WGA antibody | Vector Laboratories | AS-2024 |
| Rabbit anti-γ2 subunit antibody | Novus Biologicals | NB300-190 |
| Donkey anti-rabbit AlexaFluor 488 antibody | Invitrogen | A21206 |
| Donkey anti-rabbit AlexaFluor 594 antibody | Invitrogen | A21207 |
| Donkey anti-goat AlexaFluor 488 antibody | Invitrogen | A11055 |
| Goat anti-rabbit AlexaFluor 405 antibody | Invitrogen | A31556 |
| Rabbit anti-SK3 antibody | Alomone Labs | APC-025 |
| Rabbit anti-β-actin antibody | Bioss | bs-0061R |
| Goat anti-rabbit HRP conjugate secondary antibody | Bioss | bs-0295G |
| **Chemicals, Peptides, and Recombinant Proteins** |  |  |
| Tamoxifen | Sigma | T5648 |
| TTX | Tocris | 1078 |
| 4-AP | Tocris | 0940 |
| CNQX | Tocris | 1045 |
| D-AP5 | Tocris | 0106 |
| bicuculline | Tocris | 0131 |
| SB242084 | Tocris | 2901 |
| SB224289 | Tocris | 1221 |
| Red RetroBeads IX | Lumafluor | N/A |
| Green RetroBeads IX | Lumafluor | N/A |
| Purple RetroBeads IX | Lumafluor | N/A |
| **Experimental Models: Organisms/Strains** |  |  |
| Mouse: TPH2-CreER | Jackson Laboratory | 016584 |
| Mouse: POMC-CreER | [Berglund et al., 2013](#_ENREF_2) | N/A |
| Mouse: AgRP-Cre | [Tong et al., 2008](#_ENREF_42) | N/A |
| Mouse: DAT-CreER | Jackson Laboratory | 016583 |
| Mouse: Rosa26-tdTOMATO | Jackson Laboratory | 007905 |
| Mouse: γ2^fl/fl^ | Schweizer et al. 2003 | N/A |
| Mouse: SK3^fl/fl^ | Jackson Laboratory | 019083 |
| **Recombinant DNA** |  |  |
| AAV9-CBA-DIO-WGA-zsGreen | Dr. Richard Palmiter | N/A |
| Ad-iN/WED | Leinninger, et al. 2011 | N/A |
| pAAV9.Syn.Flex.GCaMP6m WPRE.SV40 | Addgene | 100838-AAV9 |
| AAV8-EF1α-DIO hChR2(H134R)-EYFP | UNC Gene Therapy Center | AV4378G |

1. Xu P, He Y, Cao X, Valencia-Torres L, Yan X, Saito K *et al.* Activation of Serotonin 2C Receptors in Dopamine Neurons Inhibits Binge-like Eating in Mice. *Biol Psychiatry* 2017; **81**(9)**:** 737-747.

2. Cao X, Xu P, Oyola MG, Xia Y, Yan X, Saito K *et al.* Estrogens stimulate serotonin neurons to inhibit binge-like eating in mice. *J Clin Invest* 2014; **124**(10)**:** 4351-4362.

3. Qu N, He Y, Wang C, Xu P, Yang Y, Cai X *et al.* A POMC-originated circuit regulates stress-induced hypophagia, depression, and anhedonia. *Mol Psychiatry* 2019.

**Supplemental Figures**

**
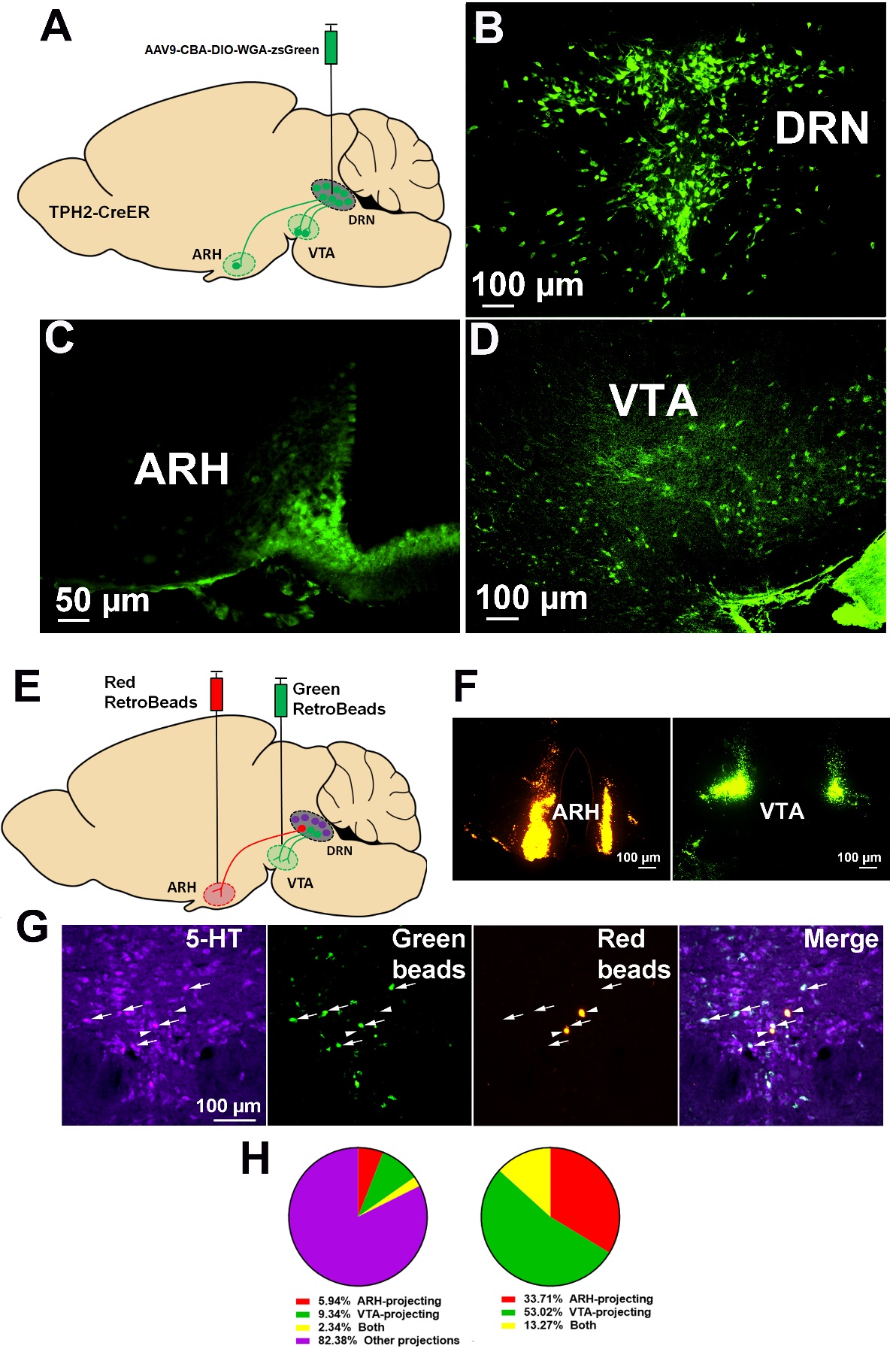
**

**Figure S1 (related to Figure 1 and 2). 5-HT^DRN^ neurons project to the ARH and VTA.** (A) Schematic illustration to stereotaxically inject AAV9-CBA-DIO-WGA-zsGreen virus into the DRN of TPH2-CreER mice for anterograde tracing from 5-HT^DRN^ neurons. (B-D) Representative microscopic images showing WGA in the DRN (B), ARH (C) and VTA (D). ARH, arcuate nucleus of hypothalamus; DRN, dorsal Raphe nuclei; VTA, ventral tegmental area. (E) Schematic illustration to stereotaxically inject Red RetroBeads into the ARH and Green Retrograde Beads into the VTA for retrograde tracing to 5-HT^DRN^ neurons. (F) Representative microscopic images showing red beads injected in the ARH and green beads injected in the VTA. Scale bar=100 µm. (G) Representative microscopic images showing 5-HT immunoreactivity (purple), red beads and green beads in the DRN. Scale bar=100 µm. Arrowheads point to 5-HT neurons co-labelled by red beads and arrows point to 5-HT neurons co-labelled by green beads. (H) Composition of 5-HT^DRN^ neurons that project to the VTA only (labelled by green beads), to ARH only (labelled by green beads) or to both within all 5-HT^DRN^ neurons (left) or within VTA- or ARH-projecting 5-HT^DRN^ neurons (right). Results are shown as mean±SEM (N=10 mice).

**
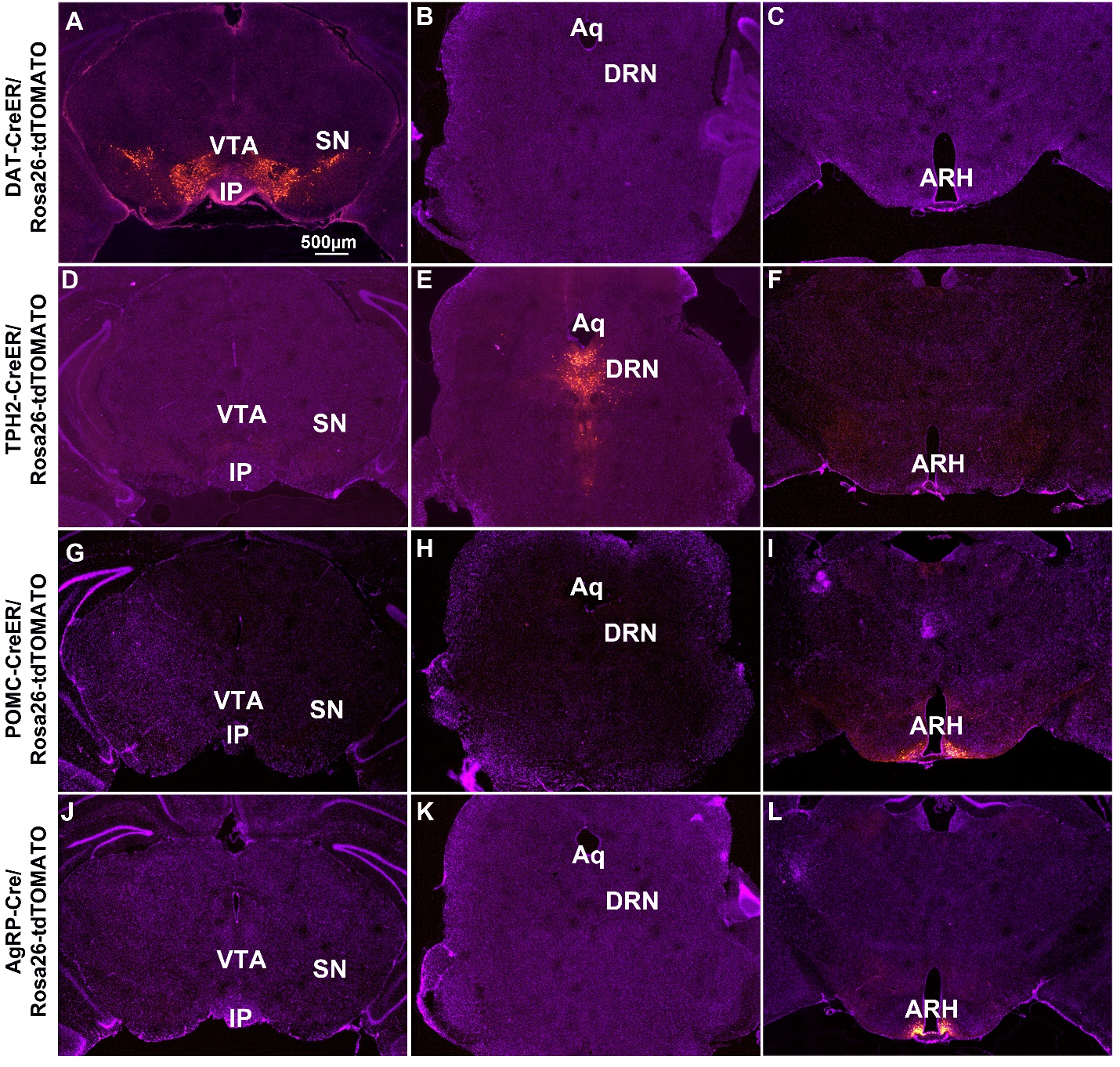
**

**Figure S2 (related to Figure 1 and 2). Distribution of Cre activity in 4 mouse lines.** (A-C) Representative microscopic images showing the presence or absence of tdTOMATO signals (red) in the VTA, SN (A), DRN (B) and ARH (C) in DAT-CreER/Rosa26-tdTOMATO mice. (D-F) Representative microscopic images showing the presence or absence of tdTOMATO signals (red) in the VTA, SN (D), DRN (E) and ARH (F) in TPH2-CreER/Rosa26-tdTOMATO mice. (G-I) Representative microscopic images showing the presence or absence of tdTOMATO signals (red) in the VTA, SN (G), DRN (H) and ARH (I) in POMC-CreER/Rosa26-tdTOMATO mice. (J-L) Representative microscopic images showing the presence or absence of tdTOMATO signals (red) in the VTA, SN (J), DRN (K) and ARH (L) in AgRP-Cre/Rosa26-tdTOMATO mice. Scale bars=500 µm. Aq, aqueduct; ARH, arcuate nucleus of hypothalamus; DRN, dorsal Raphe nuclei; IP, interpeduncular nucleus; SN, substantia nigra; VTA, ventral tegmental area.

**
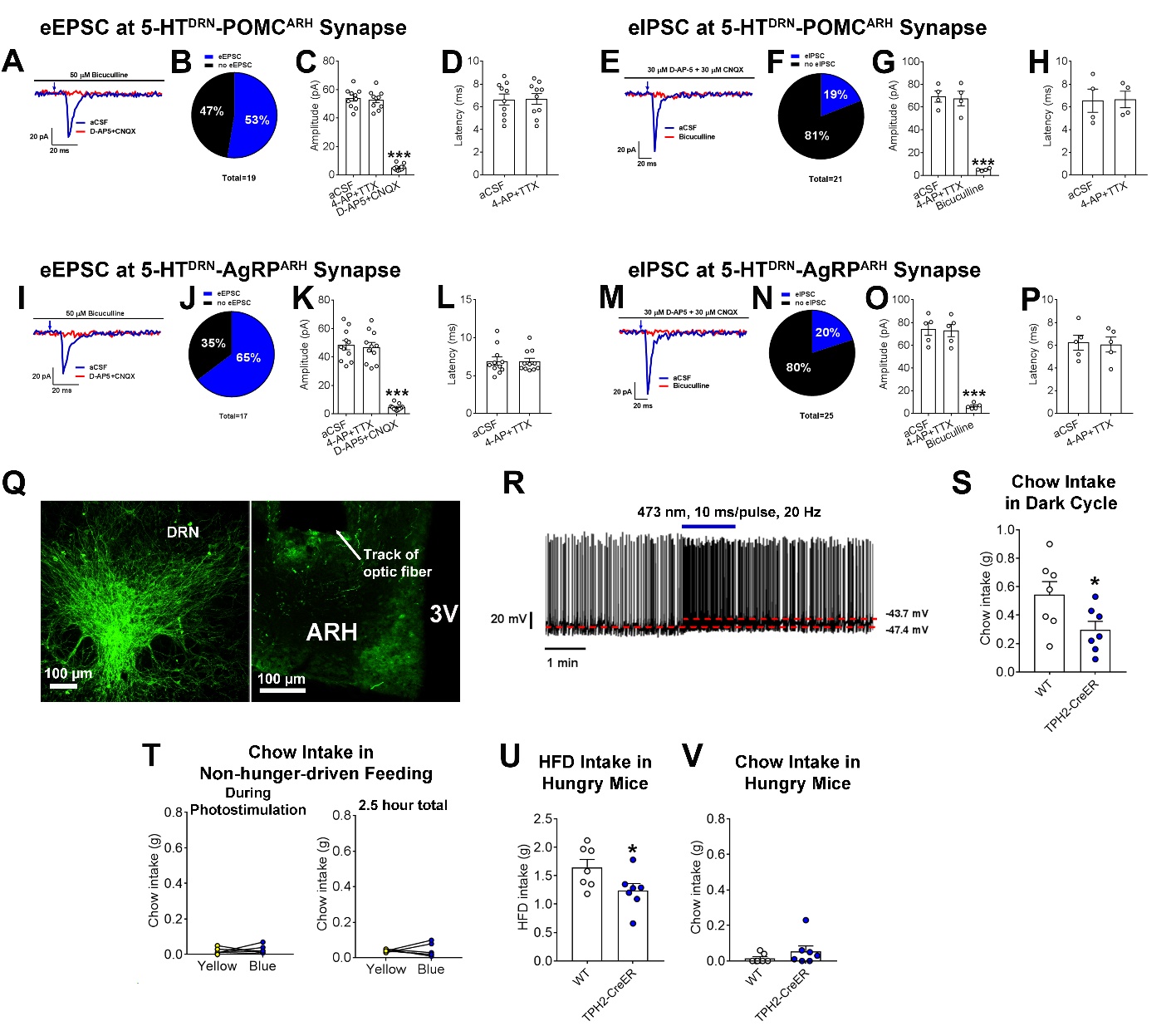
**

**Figure S3 (related to Figure 1). 5-HT^DRN^🡪ARH circuit inhibits hunger-driven feeding but not non-hunger feeding.** (A-D) Blue light-evoked EPSC (eEPSC) at the 5-HT^DRN^🡪POMC^ARH^ synapse. (A) Representative eEPSC traces in the absence or the presence of 30 µM CNQX and 30 µM D-AP5 in a bath solution containing 50 µM bicuculline. (B) Composition of 5-HT^DRN^-innervated POMC^ARH^ neurons showing eEPSC or no eEPSC (N=19 neurons from 3 mice). (C-D) Averaged amplitude (C) and latency (D) of eEPSC. Results are shown as mean±SEM. ***, P＜0.001 vs. aCSF in one-way ANOVA analyses followed by Sidak post hoc test (N=10 neurons from 3 mice). (E-H) Blue light-evoked IPSC (eIPSC) at the 5-HT^DRN^🡪POMC^ARH^ synapse. (E) Representative eIPSC traces in the absence or the presence of 50 µM bicuculline in a bath solution containing 30 µM CNQX and 30 µM D-AP5. (F) Composition of 5-HT^DRN^-innervated POMC^ARH^ neurons showing eIPSC or no eIPSC (N=21 neurons from 3 mice). (G-H) Averaged amplitude (G) and latency (H) of eIPSC. Results are shown as mean±SEM. ***, P＜0.001 vs. aCSF in one-way ANOVA analyses followed by Sidak post hoc test (N=4 neurons from 3 mice). (I-L) Blue light-evoked EPSC (eEPSC) at the 5-HT^DRN^🡪AgRP^ARH^ synapse. (I) Representative eEPSC traces in the absence or the presence of 30 µM CNQX and 30 µM D-AP5 in a bath solution containing 50 µM bicuculline. (J) Composition of 5-HT^DRN^-innervated AgRP^ARH^ neurons showing eEPSC or no eEPSC (N=17 neurons from 3 mice). (K-L) Averaged amplitude (K) and latency (L) of eEPSC. Results are shown as mean±SEM. ***, P＜0.001 vs. aCSF in one-way ANOVA analyses followed by Sidak post hoc test (N=11 neurons from 3 mice). (M-P) Blue light-evoked IPSC (eIPSC) at the 5-HT^DRN^🡪 AgRP^ARH^ synapse. (M) Representative eIPSC traces in the absence or the presence of 50 µM bicuculline in a bath solution containing 30 µM CNQX and 30 µM D-AP5. (N) Composition of 5-HT^DRN^-innervated AgRP^ARH^ neurons showing eIPSC or no eIPSC (N=25 neurons from 3 mice). (O-P) Averaged amplitude (O) and latency (P) of eIPSC. Results are shown as mean±SEM. ***, P＜0.001 vs. aCSF in one-way ANOVA analyses followed by Sidak post hoc test (N=5 neurons from 3 mice). (Q) Representative microscopic images showing ChR2-EYFP-labelled cell bodies in the DRN and fiber/terminals in the ARH. The track of optic fiber was also indicated. Scale bars=100 µm. 3V, 3^rd^ ventricle; Aq, aqueduct; ARH, arcuate nucleus of hypothalamus; DRN, dorsal Raphe nuclei. (R) Representative action potential traces validating activation of ChR2-EYFP-labelled 5-HT^DRN^ neurons in response to blue light pulses. (S) Chow intake during 6-8 pm in WT or TPH2-CreER mice fed ad libitum with the 5-HT^DRN^🡪ARH projections stimulated. Results are shown as mean±SEM individual data points. *, P＜0.05 in two-tailed unpaired t-test (N=7 mice per group). (T) Chow intake during photostimulation and during the total 2.5-hour period during the non-hunger feeding paradigm in mice with the 5-HT^DRN^🡪ARH projections unstimulated (yellow) or activated (blue). Results are shown as individual data points (N=6 mice per group). Note that the number of data points may appear fewer than the N values because multiple mice showed very small and similar amount of chow intake and therefore the data points partially overlapped. (U-V) HFD intake (U) and chow intake (V) in the morning after an overnight fasting in WT or TPH2-CreER mice fed ad libitum with the 5-HT^DRN^🡪ARH projections stimulated. Results are shown as mean±SEM individual data points. *, P＜0.05 in two-tailed unpaired t-test (N=7 mice per group).


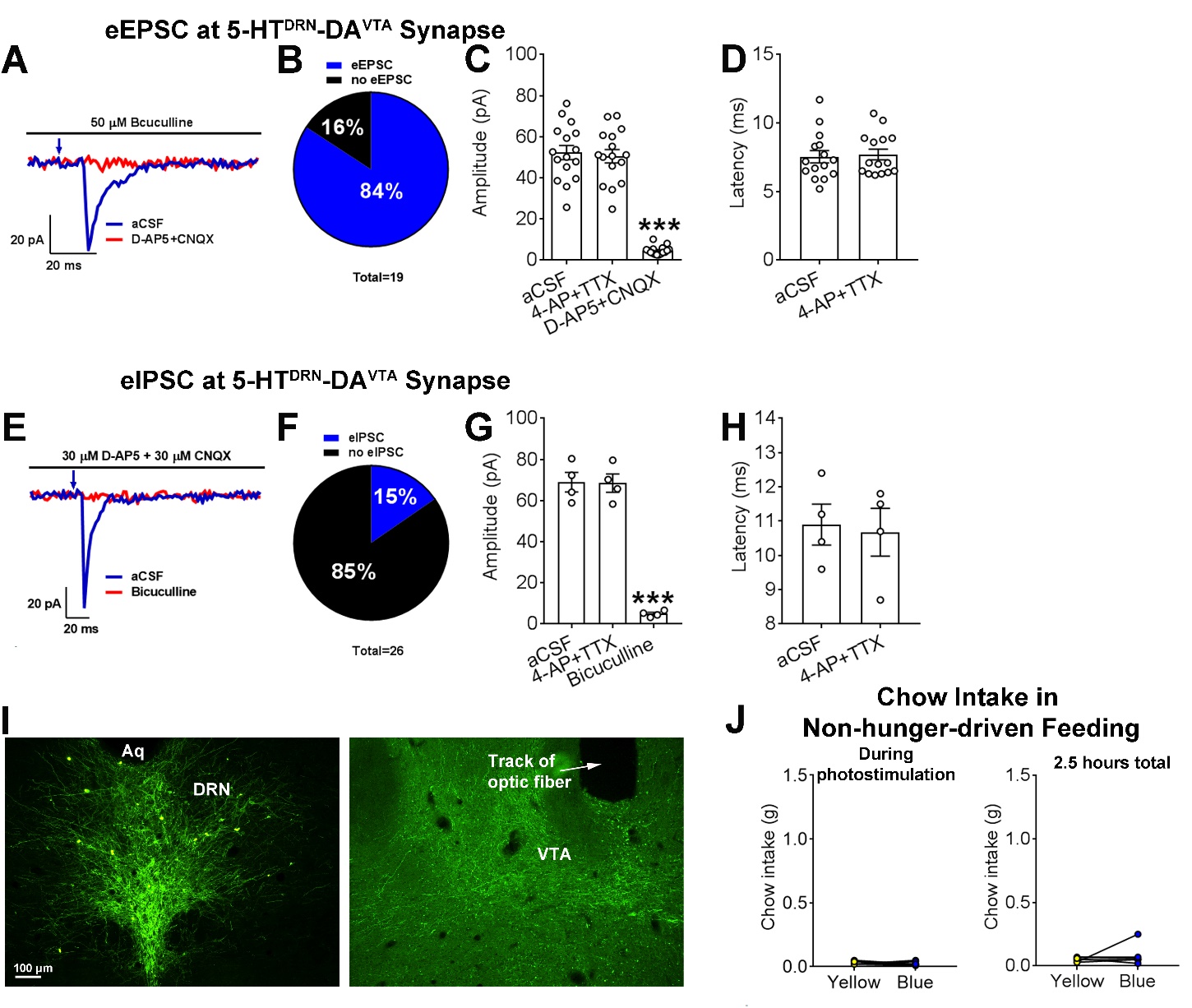


**Figure S4 (related to Figure 2). 5-HT^DRN^🡪VTA circuit inhibits non-hunger feeding but not hunger-driven feeding.** (A-D) Blue light-evoked EPSC (eEPSC) at the 5-HT^DRN^🡪DA^VTA^ synapse. (A) Representative eEPSC traces in the absence or the presence of 30 µM CNQX and 30 µM D-AP5 in a bath solution containing 50 µM bicuculline. (B) Composition of 5-HT^DRN^-innervated DA^VTA^ neurons showing eEPSC or no eEPSC (N=19 neurons from 3 mice). (C-D) Averaged amplitude (C) and latency (D) of eEPSC. Results are shown as mean±SEM. ***, P＜0.05 vs. aCSF in one-way ANOVA analyses followed by Sidak post hoc test (N=16 neurons from 3 mice). (E-H) Blue light-evoked IPSC (eIPSC) at the 5-HT^DRN^🡪 DA^VTA^ synapse. (E) Representative eIPSC traces in the absence or the presence of 50 µM bicuculline in a bath solution containing 30 µM CNQX and 30 µM D-AP5. (F) Composition of 5-HT^DRN^-innervated DA^VTA^ neurons showing eIPSC or no eIPSC (N=26 neurons from 3 mice). (G-H) Averaged amplitude (G) and latency (H) of eIPSC. Results are shown as mean±SEM. ***, P＜0.05 vs. aCSF in one-way ANOVA analyses followed by Sidak post hoc test (N=4 neurons from 3 mice). (I) Representative microscopic images showing ChR2-EYFP-labelled cell bodies in the DRN and fiber/terminals in the VTA. The track of optic fiber was also indicated. Scale bars=100 µm. Aq, aqueduct; DRN, dorsal Raphe nuclei; VTA, ventral tegmental area. (J) Chow intake during photostimulation and during the total 2.5-hour period during the non-hunger feeding paradigm in mice with the 5-HT^DRN^🡪VTA projections unstimulated (yellow) or activated (blue). Results are shown as individual data points (N=6 mice per group). Note that the number of data points may appear fewer than the N values because multiple mice showed very small and similar amount of chow intake and therefore the data points partially overlapped.


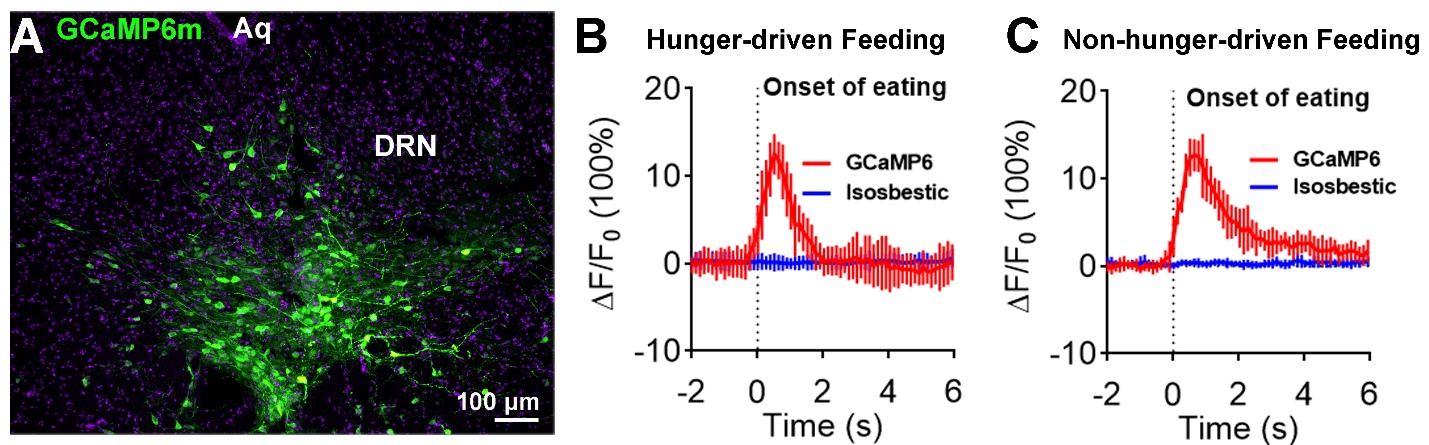


**Figure S5 (related to Figure 3). Dynamics of 5-HT^DRN^ neuron activity during hunger-driven feeding and non-hunger feeding.** (A) A representative microscopic image showing GCaMP6m-labelled 5-HT^DRN^ neurons. Scale bar=100 µm. Aq, aqueduct; DRN, dorsal Raphe nuclei. (B-C) Averaged GCaMP6 and isosbestic signals in 5-HT^DRN^ neurons associated with eating bouts during hunger-driven feeding (B) or non-hunger feeding (C). Results are shown as mean±SD (N=11 or 20 events).


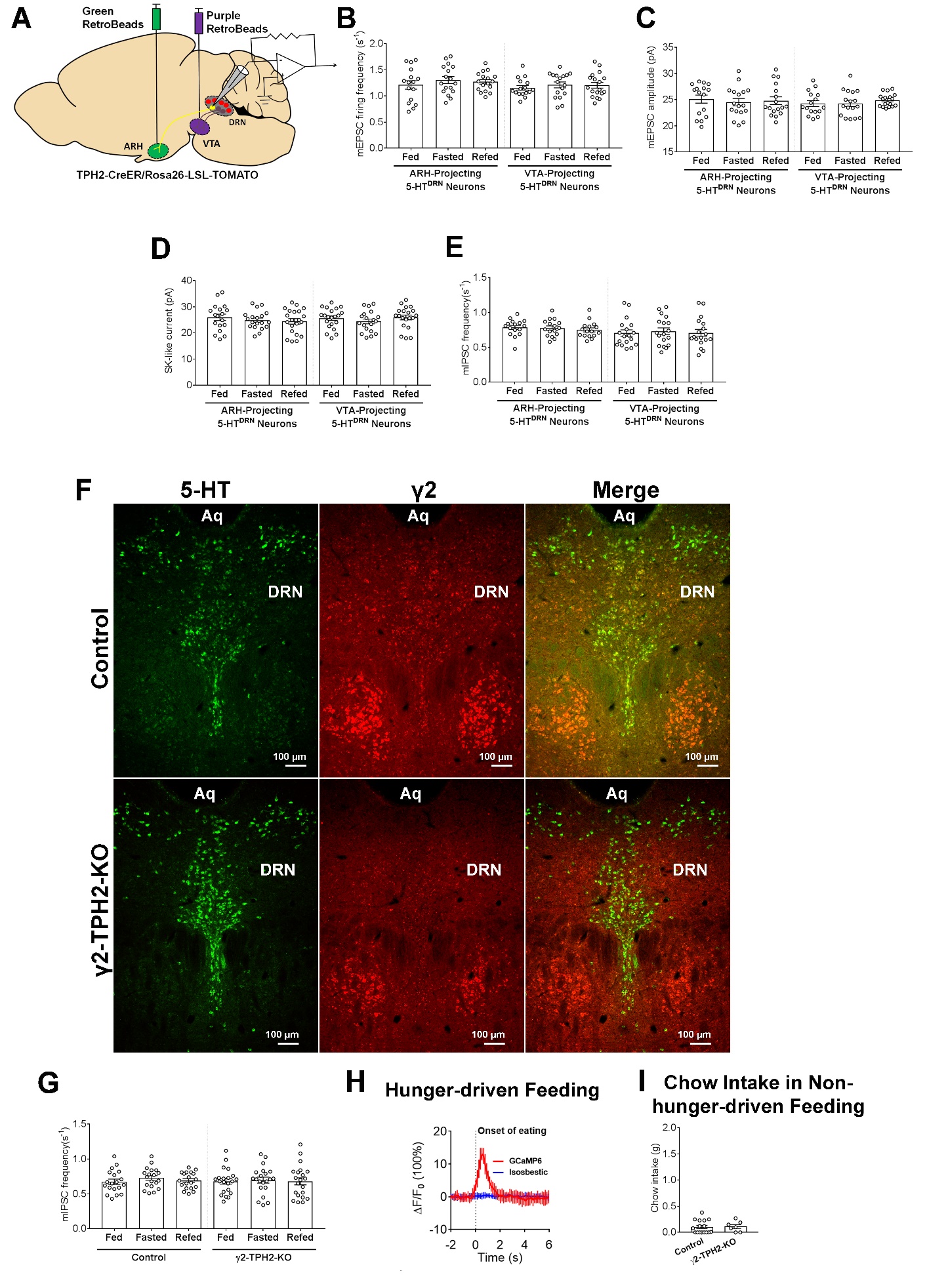


**Figure S6. GABA_A_ currents mediate dynamic changes in ARH-projecting 5-HT^DRN^ neuron activity during hunger-driven feeding.** (A) Schematic illustration for electrophysiological recordings of ARH-projecting and VTA-projecting 5-HT^DRN^ neurons. (B-C) Frequency (B) and amplitude (C) of mEPSC in ARH-projecting or VTA-projecting 5-HT^DRN^ neurons in mice fed ad libitum, fasted for overnight, or fasted for overnight followed by 2-hour refeeding. Results are shown as mean±SEM with individual data points (N=16-18 neurons from 3 mice per group). (D) Amplitude of SK-like currents in ARH-projecting or VTA-projecting 5-HT^DRN^ neurons in mice fed ad libitum, fasted for overnight, or fasted for overnight followed by 2-hour refeeding. Results are shown as mean±SEM with individual data points (N=18-21 neurons from 3 mice per group). (E) Frequency of mEPSC in ARH-projecting or VTA-projecting 5-HT^DRN^ neurons in mice fed ad libitum, fasted for overnight, or fasted for overnight followed by 2-hour refeeding. Results are shown as mean±SEM with individual data points (N=16-18 neurons from 3 mice per group). (F) Immunofluorescence for 5-HT (green), GABA_A_ γ2 subunit (red) and merge in the DRN in control or γ2-TPH2-KO mice. Scale bar=100 µm. Aq, aqueduct; DRN, dorsal Raphe nuclei. (G) Frequency of mIPSC in ARH-projecting 5-HT^DRN^ neurons in control or γ2-TPH2-KO mice fed ad libitum, fasted for overnight, or fasted for overnight followed by 2-hour refeeding. Results are shown as mean±SEM with individual data points (N=14-25 neurons from 3 mice per group). (H) Averaged GCaMP6 and isosbestic signals in 5-HT^DRN^ neurons in γ2-TPH2-KO mice associated with eating bouts during hunger-driven feeding. Results are shown as mean±SD (N=14 events). (I) The amount of chow intake during 2.5-hour non-hunger feeding in control or γ2-TPH2-KO mice. Results are shown as mean±SEM with individual data points (N=7 or 16 mice per group).


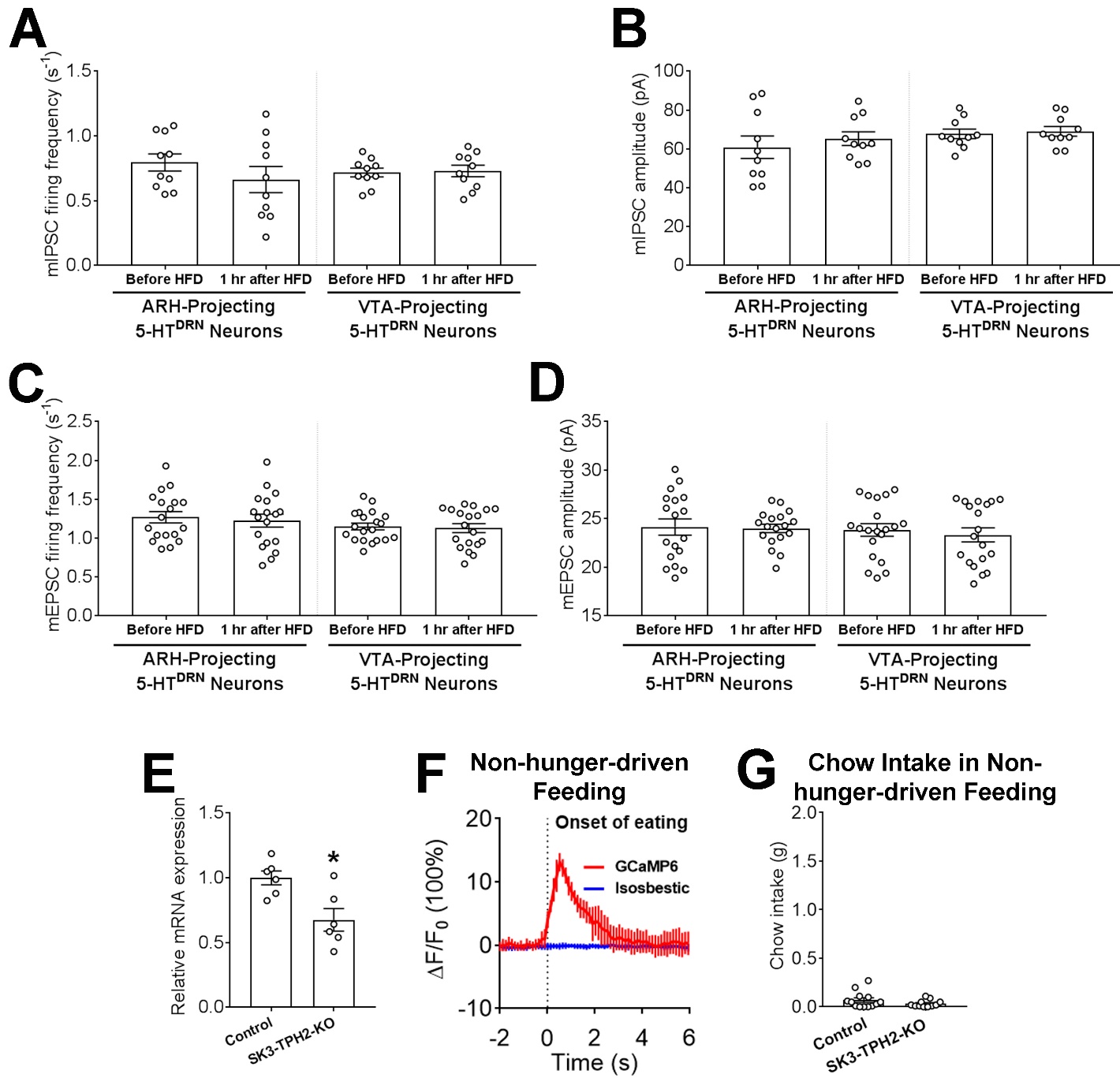


**Figure S7. SK currents mediate dynamic changes in VTA-projecting 5-HT^DRN^ neuron activity during non-hunger feeding.** (A-B) Frequency (A) and amplitude (B) of mIPSC in ARH-projecting or VTA-projecting 5-HT^DRN^ neurons in mice before and 1-hour after non-hunger feeding. Results are shown as mean±SEM with individual data points (N=10 neurons from 3 mice per group). (C-D) Frequency (C) and amplitude (D) of mEPSC in ARH-projecting or VTA-projecting 5-HT^DRN^ neurons in mice before and 1-hour after non-hunger feeding. Results are shown as mean±SEM with individual data points (N=18-20 neurons from 3 mice per group). (E) Relative mRNA levels of SK3 in the DRN of control or SK3-TPH2-KO mice. Results are shown as mean±SEM with individual data points. *, P<0.05 in t-test (N=6 mice per group). (F) Averaged GCaMP6 and isosbestic signals in 5-HT^DRN^ neurons in SK3-TPH2-KO mice associated with eating bouts during non-hunger feeding. Results are shown as mean±SD (N=14 events). (G) The amount of chow intake during 2.5-hour non-hunger feeding in control or SK3-TPH2-KO mice. Results are shown as mean±SEM with individual data points (N=11 or 13 mice per group).


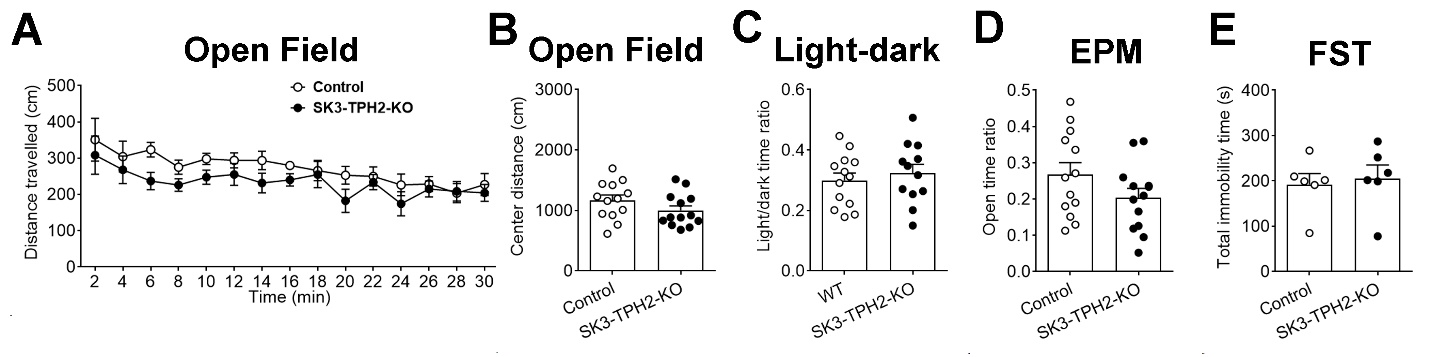


**Figure S8. Behaviors in SK3-TPH2-KO mice.** Locomotion, anxiety-like and depression-like behaviors were examined in the open field tests (A-B), in light-dark test (C), in elevated plus maze test (D), and in forced swim test (E) in male WT and SK3-TPH2-KO mice. Results are shown as mean±SEM with individual data points. *, P<0.05 in two-tailed unpaired t-test (N=6-18 mice per group).


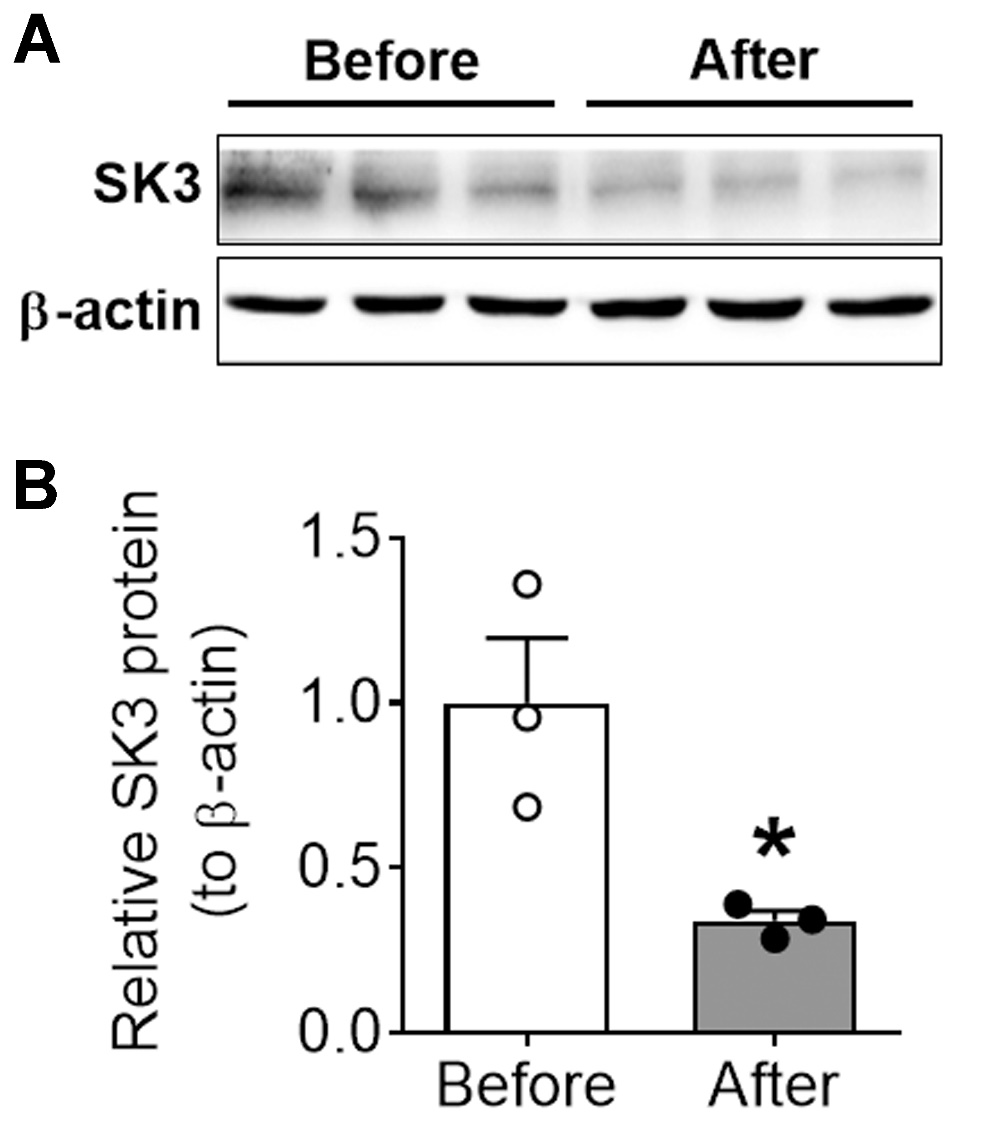


**Figure S9. Regulations of SK3 protein levels.** (A) Western Blotts showing expression of SK3 and β-actin protein in the DRN of mice that were euthanized immediately before the non-hunger feeding and at 2.5 hours after the non-hunger feeding started. (B) Quantification for the relative SK3 protein levels in the DRN. Results are shown as mean±SEM with individual data points. *, P<0.05 in two-tailed unpaired t-test (N=3 mice per group).
